# RAG: a regularized adaptive graph-based method for rare-cell identification from single-cell expression data

**DOI:** 10.1093/bib/bbag379

**Published:** 2026-07-17

**Authors:** Xingsu Wang, Yanyan Chen, Dian Huang, Zhen Ju, Qi Wei, Shu Li, Shengzhong Feng

**Affiliations:** College of Applied Sciences, Macau Polytechnic University, Gomes Street, Macau 999078, China; Laboratory of High-Performance Intelligent Computing, Guangdong Institute of Intelligence Science and Technology, Building 6, 2515 North Huandao Road, Hengqin Guangdong-Macao In-Depth Cooperation Zone, Zhuhai, Guangdong 519031, China; Department of Oncology, Jiangsu Cancer Hospital, 42th, Baiziting Road, Nanjing, Jiangsu 230031, China; Laboratory of High-Performance Intelligent Computing, Guangdong Institute of Intelligence Science and Technology, Building 6, 2515 North Huandao Road, Hengqin Guangdong-Macao In-Depth Cooperation Zone, Zhuhai, Guangdong 519031, China; Laboratory of High-Performance Intelligent Computing, Guangdong Institute of Intelligence Science and Technology, Building 6, 2515 North Huandao Road, Hengqin Guangdong-Macao In-Depth Cooperation Zone, Zhuhai, Guangdong 519031, China; Laboratory of High-Performance Intelligent Computing, Guangdong Institute of Intelligence Science and Technology, Building 6, 2515 North Huandao Road, Hengqin Guangdong-Macao In-Depth Cooperation Zone, Zhuhai, Guangdong 519031, China; College of Applied Sciences, Macau Polytechnic University, Gomes Street, Macau 999078, China; Laboratory of High-Performance Intelligent Computing, Guangdong Institute of Intelligence Science and Technology, Building 6, 2515 North Huandao Road, Hengqin Guangdong-Macao In-Depth Cooperation Zone, Zhuhai, Guangdong 519031, China

**Keywords:** rare-cell identification, single-cell RNA sequencing, regularized adaptive graph

## Abstract

Rare-cell identification is essential for dissecting disease mechanisms and developmental programs. Existing methods mostly rely on fixed-size neighbourhood graphs to separate rare-cell populations in single-cell expression data, which may embed rare cells into dominant clusters under varying sampling densities. This paper proposes the RAG method for identifying rare cells based on regularized adaptive graphs, which can better separate rare cells. Specifically, the regularized adaptive graph is constructed by estimating cell-specific radii from Euclidean–cosine hybrid dissimilarity to constrain effective neighbours and stabilize the adjacency, and then, assigning locally scaled hybrid affinities to make affinity magnitudes comparable across density-varying regions. Across 10 real single-cell RNA sequencing datasets, RAG overall outperformed six state-of-the-art methods, improving precision, F1 score, and rare-type coverage rate over the second-ranked baseline by 42%, 26%, and 35%, respectively. A case study on colorectal tumour tissue shows that RAG is more accurate in recovering annotated rare-cell populations and separating the substructure from the major population than the other evaluated methods. Further analyses on mouse airway epithelium and two pancreas datasets showed that about half of RAG-resolved small clusters corresponded to known annotated populations or marker-supported subpopulations.

The source code is available at https://github.com/wangxingsu/RAG.

## Introduction

Identifying rare cells is crucial for dissecting disease mechanisms and developmental programs [1, 2]. For example, the activation of rare immune cells can trigger immune responses [3, 4], rare drug-resistant subpopulations in tumours could lead to treatment failure [5, 6], and rare precursor cells may govern differentiation trajectories and functional phenotypes [7]. Based on transcriptome-wide gene expression profiles from (f) sequencing (scRNA-seq), rare-cell identification is feasible [8, 9]. However, it remains challenging as rare populations are undersampled and may be absorbed into dominant populations during general single-cell analysis [10, 11]. Many methods have been developed to facilitate rare-cell identification by refining representation learning and clustering strategies [12].

Existing rare-cell identification methods can be broadly classified into three categories. The first category consists of feature-driven methods, such as SCA [13], the GiniClust series [14–16], and CIARA [17]. SCA [13] performs dimension reduction using surprisal scores to highlight locally unexpected genes, enhancing the separation of rare cells. The GiniClust series [14–16] uses Leiden clustering on Gini-index and Fano-factor features to improve cell identification performance and efficiency. CIARA [17] uses $k$-nearest neighbours ($k$NN) enrichment testing based on the hypergeometric distribution for locally expressed rare-cell markers. The second category centres on recursive clustering with outlier detection for rare-cell populations, including RaceID [18, 19], scCAD [20], and CellSIUS [21]. RaceID [18, 19] detects expression outliers using a noise model after coarse clustering, then reclusters them into subpopulations for rare-cell discovery. The scCAD [20] iteratively decomposes clusters based on the most differential signals and uses anomaly detection to identify rare cells. CellSIUS [21] detects bimodal, cluster-specific subgroups within coarse clusters and reclusters their outlier cells into rare subpopulations. The third category analyses rare populations using density-based methods like FiRE [22], GapClust [23], and EDGE [24]. FiRE [22] estimates approximate densities from sketches to rapidly assign continuous rarity scores to cells. GapClust [23] detects rare cells using a second-order statistic on local distance curves, capturing gap patterns with a lightweight and sensitive design. EDGE [24] ensembles sketch-based weak learners built from random genes to form a similarity matrix, yielding embeddings to separate rare populations.

Though these methods have shown promising results in specific settings, their performance remains limited. Many feature-driven methods rely on $k$NN graphs to estimate local expression patterns and perform downstream clustering. Yet, fixed-size neighbourhoods may not adapt to uneven cell density distributions in feature space, leading to unstable boundaries for rare populations. Recursive clustering-based methods with outlier detection depend on the quality of the initial partition and may struggle to decide when to stop decomposing or merging, leading to over- or under-clustering and increasing runtime as the refinement proceeds. Density-based methods focus on rarity scoring and distance- or sketch-based similarity estimation; without a dedicated mechanism to explicitly enhance rare-type expression deviations during representation learning, identifying rare populations may remain challenging in complex tissues.

Here, we propose RAG, a regularized adaptive graph-based framework for rare-cell identification. RAG follows a three-stage single-cell analysis pipeline, including preprocessing, representation learning, and clustering. In the representation learning and clustering stages, RAG uses the RAG operator to construct regularized adaptive graphs. The RAG operator first constructs candidates for each cell by taking the union of Euclidean and cosine nearest neighbours to preserve both magnitude-sensitive proximity and direction-sensitive expression-pattern similarity. It then uses cell-specific radii based on hybrid dissimilarity to constrain effective neighbours and stabilize the adjacency. Lastly, it assigns affinities to retained edges in a locally scaled hybrid space, which normalizes edge magnitudes across data with different sampling densities. By the RAG operator, RAG preserves informative local contexts for rare cells during representation learning and provides a refined affinity graph for downstream community detection.

We compared RAG with state-of-the-art methods for rare-cell identification. On *in vivo* mixture datasets and 10 real scRNA-seq datasets, RAG achieves the highest overall precision. As the number of rare cells increases, RAG reaches reliable identification at lower rarity levels and recovers more rare types across worst-case scenarios, indicating improved robustness. Experiments on large datasets show that RAG is faster than the most accurate method and has a near-linear runtime. In addition, a colorectal tumour tissue case study shows that RAG not only accurately recovers annotated rare cell types but also resolves subtle substructure within major cell populations, providing clues to understanding the tumour microenvironment and disease progression.

## Methods

### Overview

**Figure 1:**
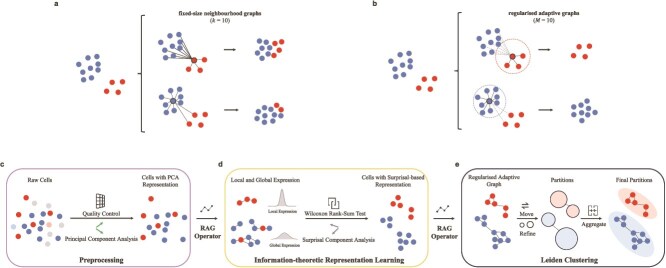
Overview of RAG. Points denote dominant and rare cells, and dashed circles indicate cell-specific radius. (a) A fixed-size neighbourhood graph selects a fixed number of nearest cells as candidate neighbours. (b) A regularized adaptive graph, constructed by averaging hybrid dissimilarities among candidate neighbours to define the cell-specific radius, retains effective neighbours. (c) Standard preprocessing, including quality control and principal component analysis, produces a PCA representation of cells. (d) With the input of PCA representation, RAG operator constructs the first regularized adaptive graph and derives neighbourhoods for Wilcoxon-based surprisal component analysis, yielding an information-theoretic representation. (e) With the input of information-theoretic representation, the RAG operator constructs the second regularized adaptive graph for Leiden community detection.

The RAG process can be divided into three parts: preprocessing, representation learning, and clustering. In the representation learning and clustering parts, the information-theoretic representation learning method proposed by SCA [13] and the Leiden clustering [25] are adopted, respectively. On this basis, RAG developed a regularized adaptive graph that adaptively selects cell-specific neighbourhoods rather than a fixed size, as detailed in Fig. 1a and b. Given an input expression matrix, RAG performs preprocessing, learns an information-theoretic representation using a neighbourhood from the first regularized adaptive graph, and then runs Leiden community detection on a second regularized adaptive graph to output cluster assignments, as shown in Fig. 1c–e. The RAG operator is the core construction module that generates these regularized adaptive graphs.

### Regularized adaptive graph construction

We use the RAG operator to construct a regularized adaptive graph. The core idea is to estimate a cell-specific radius from candidate neighbours in the hybrid dissimilarity space to control adjacency, pruning distant neighbours beyond this radius while retaining local edges. The Euclidean distance captures magnitude-sensitive proximity, whereas the cosine distance captures direction-sensitive expression-pattern similarity [26, 27]. It then assigns locally scaled affinities to the retained edges, making affinity magnitudes comparable across data with different sampling densities.

For each cell $i$, candidate neighbours are obtained by taking the union of cells selected under the Euclidean and cosine distance criteria. The candidate-neighbour is defined as: 


(1) \begin{align*}& \mathcal{C}_{i} = \mathcal{N}^{(E)}_{i}\cup \mathcal{N}^{(C)}_{i},\end{align*}

where $\mathcal{C}_{i}$ is the candidate-neighbour set, and $\mathcal{N}^{(E)}_{i}$ and $\mathcal{N}^{(C)}_{i}$ denote the candidate cells nearest to cell $i$ under the Euclidean and cosine distance criteria, respectively. Both of the candidate cell counts are controlled by 


(2) \begin{align*}& M = \max\left(5,\left\lceil \rho_{M} \cdot N \right\rceil\right),\end{align*}

where $M$ is the number of candidate neighbours and $\rho _{M}$ is the candidate-neighbour ratio, which is set to $0.5\%$ by default. The lower bound of 5 prevents the candidate set from becoming too small on small datasets.

To make candidate cells from the Euclidean and cosine distance criteria comparable, a locally normalized hybrid dissimilarity $d^{(H)}_{i,j}$ is defined as: 


(3) \begin{align*}& {d^{(H)}_{i,j} = \alpha\frac{d^{(E)}_{i,j}}{\sigma^{(E)}_{i}} + (1-\alpha)\frac{d^{(C)}_{i,j}}{\sigma^{(C)}_{i}},}\end{align*}

where $\alpha $ balances the Euclidean component, which captures magnitude-sensitive proximity, and the cosine component, which captures direction-sensitive expression-pattern similarity. The default value of $\alpha $ is $0.5$, and $\sigma ^{(E)}_{i}$ and $\sigma ^{(C)}_{i}$ are the local Euclidean and cosine scales of cell $i$, computed from the farthest candidate under each distance criterion, respectively.

For each cell, edges are retained only for candidate neighbours within this radius. We compute the radius threshold from the candidate neighbours as follows: 


(4) \begin{align*}& {r_{i} = \frac{\sum_{j\in\mathcal{C}_{i}}d^{(H)}_{i,j}}{|\mathcal{C}_{i}|-\eta},}\end{align*}

where $r_{i}$ is the local radius of the cell $i$, $d^{(H)}_{i,j}$ denotes the hybrid dissimilarity between cells of the cell $i$ and $j$, and $\eta $ is a small bias-correction constant with a default value of $1$. The initial directed graph retains the edge from cell $i$ to cell $j$ if $j\in \mathcal{C}_{i}$ and $d^{(H)}_{i,j}\le r_{i}$.

On this graph, we assign edge weights using a locally scaled hybrid Gaussian kernel based on the self-tuning kernel [28]. We define the affinity between cells $i$ and $j$ as: 


(5) \begin{align*}& { {S}_{ij} = \exp\!\left( -\alpha\frac{\bigl(d^{(E)}_{i,j}\bigr)^{2}}{\sigma^{(E)}_{i}\sigma^{(E)}_{j}} -(1-\alpha)\frac{\bigl(d^{(C)}_{i,j}\bigr)^{2}}{\sigma^{(C)}_{i}\sigma^{(C)}_{j}} \right)\kern-2pt, }\end{align*}

where ${S}_{ij}$ is the locally scaled hybrid affinity. Here, $\sigma ^{(E)}_{i}$ and $\sigma ^{(E)}_{j}$ denote the Euclidean local scales of cells $i$ and $j$, respectively, while $\sigma ^{(C)}_{i}$ and $\sigma ^{(C)}_{j}$ denote their cosine local scales. These scales are computed as the distances to the farthest neighbours among the Euclidean and cosine candidate neighbours, respectively.

After directed candidate edges are selected and weighted by the RAG operator, the resulting directed regularized adaptive graph may still contain asymmetric edges or weak bridge-like connections that can distort community structure. We therefore apply standard graph-processing steps to obtain a conservative undirected regularized adaptive graph. The graph is first symmetrized by retaining mutually supported edges and assigning each cell pair the smaller of the two directed affinities. Edges with insufficient shared-neighbour support are then removed to reduce weak bridge-like connections. Together, these steps produce a conservative symmetric affinity matrix that better reflects the local connectivity structure on the manifold.

We construct the regularized adaptive graph using two implementation modes. For datasets with >20 000 cells, we use scikit-learn’s NearestNeighbors and a compressed sparse row matrix representation to retrieve and store Euclidean and cosine candidate neighbours. However, using sparse nearest-neighbour retrieval on small datasets can change the candidate-neighbour set and the resulting local graph structure. This effect is more visible on small datasets because a small change in candidate neighbours can affect the adaptive radius and the retained edges. To mitigate this issue, we compute the full Euclidean and cosine pairwise distance matrices exhaustively for small datasets to obtain an exact regularized adaptive graph. Although exhaustive computation incurs $\mathcal{O}(n^{2})$ memory and time, the modest dataset size keeps the absolute cost acceptable. Both constructions follow the same adaptive criteria.

Finally, we regularize the graph by controlling the adjacency and normalizing the affinity magnitude. It produces an affinity matrix that remains stable across varying sampling densities: 


(6) \begin{align*}& S = \mathrm{RAG}(Z),\end{align*}

where $Z$ and $S$ represent the input matrix and output affinity matrix of the RAG operator, respectively.

### Preprocessing

We apply common scRNA-seq preprocessing [29–31] to ensure data quality and comparability across cells. First, we remove the raw cells with zero total counts or fewer than 200 detected genes to filter the low-quality cells. Then, exclude the genes expressed in fewer than three cells to reduce extremely sparse features. Lastly, mitochondrial genes are annotated, and cells with abnormal quality metrics are removed: those in the top 5% of the mitochondrial fraction and those whose detected gene counts lie within the top or bottom 1% of the distribution. Thresholds follow common Scanpy practice and were kept constant across all datasets.

After quality control, library sizes are normalized per cell and log-transformed to stabilize variance. Genes with expression variance <0.1 were removed, and the top 5000 highly variable genes (HVGs) were selected for downstream analysis. PCA is then applied to obtain a denoised low-dimensional representation matrix $X_{\mathrm{pca}}$, with the number of components selected according to a 0.9 cumulative explained-variance threshold.

### Information-theoretic representation learning

Given a PCA representation matrix, we apply the RAG operator and compute an information-theoretic representation [13] that captures local–global expression deviations.

We first construct a regularized adaptive graph by applying the RAG operator to the PCA representation: 


(7) \begin{align*}& S1 = \mathrm{RAG}(X_{\mathrm{pca}}),\end{align*}

where $S1$ denotes the denoised regularized adaptive graph.

Then, the denoised regularized adaptive graph $S1$ can be used to construct cell neighbourhoods. For each cell, its neighbourhood is constituted of neighbours whose affinity is >0, as shown by the following: 


(8) \begin{align*}& \mathcal{N}_{i} = \{ j \mid S1_{ij}> 0 \},\end{align*}

where $\mathcal{N}_{i}$ denotes the neighbours of cell $i$, and $S1_{ij}$ is the affinity of cell $i$ to neighbour $j$.

The neighbourhood of each cell defines a local reference set, which we use to assess whether the expression of each gene within this local context deviates from its global distribution across all cells. Specifically, for cell $i$ and gene $g$, we perform a two-sided Wilcoxon rank-sum test by comparing the neighbourhood expression to the remaining cells, producing a $P$-value: 


(9) \begin{align*}& p_{ig} = \mathrm{Wilcoxon}\!\bigl( X_{\mathcal{N}_{i}}(g),\, X_{\mathrm{all}\setminus \mathcal{N}_{i}}(g) \bigr),\end{align*}

where $X_{\mathcal{N}_{i}}(g)$ denotes the expression values of gene $g$ in the neighbourhood $\mathcal{N}_{i}$ of cell $i$, and $X_{\mathrm{all}\setminus \mathcal{N}_{i}}(g)$ denotes its expression in all other cells.

Since many genes are tested for each cell, we correct for multiple testing within each cell using a family-wise error rate adjustment in the form used by SCA, yielding the corrected probability: 


(10) \begin{align*}& p_{ig}^{*} = 1 - (1 - p_{ig})^{N_{t}},\end{align*}

where $N_{t}$ is the number of tested genes. Under the null hypothesis of random gene expression, $p_{ig}^{*}$ can be interpreted as the probability that, in cell $i$, at least one gene exhibits a neighbourhood deviation at least as extreme as that observed for gene $g$.

Following the surprisal transformation, the signed surprisal score is defined as: 


(11) \begin{align*}& I_{ig} = -\log p_{ig}^{*} \cdot s_{ig},\end{align*}

where $s_{ig}$ is a sign term indicating over- or under-expression. Following the sign form in SCA, the score can be written as: 


(12) \begin{align*}& I_{ig} = -\mathrm{sgn}\!\left( \mathrm{ranksum}\bigl(g,\mathcal{N}_{i}\bigr) - \frac{|\mathcal{N}_{i}| (N-|\mathcal{N}_{i}|)}{2} \right)\, \log p_{ig}^{*},\end{align*}

where $\mathrm{ranksum}(g,\mathcal{N}_{i})$ denotes the Wilcoxon rank-sum statistic computed for gene $g$ between $\mathcal{N}_{i}$ and its complement. $N$ denotes the total number of cells and $|\mathcal{N}_{i}|$ denotes the cell $i$ neighbourhood sample size. Collecting all $I_{ig}$ produces the surprise matrix $I$.

Based on the surprisal matrix, SCA seeks linear combinations of genes whose surprisal scores have maximal magnitude across cells. This corresponds to computing the leading right singular vectors of the surprisal matrix by performing singular value decomposition [32]: 


(13) \begin{align*}& I = U\Sigma V^{\top},\end{align*}

where the columns of $V$ are the right singular vectors.

The final representation is obtained by projecting the expression matrix onto these loadings: 


(14) \begin{align*}& X_{\mathrm{sca}} = X V_{:,1:D},\end{align*}

where $X$ denotes the log-normalized HVG expression matrix, yielding an $N \times D$ low-dimensional, information-theoretic representation of the $N$ cells that emphasizes genes whose local behaviour deviates most from the global background. The first $D$ loading vectors $V_{:,1:D}$ define the metagenes, with $D$ set to 50 by default.

### Clustering

This stage applies the RAG operator to the information-theoretic representation, constructing the second regularized adaptive graph, which is used for Leiden clustering [25] to generate cell populations: 


(15) \begin{align*}& S2 = \mathrm{RAG}(X_{\mathrm{sca}}),\end{align*}

where $S2$ denotes the surprisal-based regularized adaptive graph.

This graph encodes refined local structure and provides an affinity adjacency for Leiden community detection. We partition the RAG-defined regularized adaptive graph using the Leiden algorithm, which maximizes modularity: 


(16) \begin{align*}& Q = \frac{1}{2m} \sum_{i,j} \Bigl[ S2_{ij} - \gamma\,\frac{k_{i} k_{j}}{2m} \Bigr]\, \mathbb{I}(c_{i}=c_{j}),\end{align*}

where $m=\tfrac 12\sum _{i,j} S2_{ij}$ is the total weight, $\gamma $ is the resolution parameter default as 1, $k_{i}=\sum _{j} S2_{ij}$ is the node strength, $c_{i}$ denotes the cluster label of cell $i$, and $\mathbb{I}(c_{i}=c_{j})$ equals 1 if two cells are assigned to the same cluster and 0 otherwise.

## Materials

### Datasets

We evaluated RAG on 11 scRNA-seq datasets, as detailed in Table 1. Specifically, to enable direct, reproducible comparisons under widely accepted settings, we surveyed the 18 most highly cited rare-cell identification papers published in the past decade (Google Scholar) [12–18, 20–24, 38–44] and selected the eight datasets that are most frequently used, publicly available, and provide curated cell-type annotations. These benchmarks cover *in vitro* mixtures, immune populations, and diverse healthy tissues from both human and mouse, spanning developmental, epithelial, neural, and organ-specific systems. In addition, we included three nonpublic mouse colorectal tumour datasets to assess performance in tumour microenvironment scenarios.

**Table 1:** Summary of the scRNA-seq datasets.

Name	Species	Tissues	Accession	Cells	Genes	Types	Rare types
Airway [33]	Mouse	Tracheal epithelium	GSE103354	7193	18 388	7	4
Deng [34]	Mouse	Embryo	GSE45719	259	22 431	10	5
Hrvatin [35]	Mouse	Visual cortex	GSE102827	48 266	25 187	8	2
Intestinal organoid [18]	Mouse	Intestinal crypts	GSE62270	238	23 538	7	4
CRC metastases G1	Mouse	Metastasis tumour	Nonpublic	27 201	12 000	8	4
CRC metastases G2	Mouse	Metastasis tumour	Nonpublic	26 592	12 000	8	5
CRC metastases G3	Mouse	Metastasis tumour	Nonpublic	20 740	12 000	8	4
Pancreas [36]	Human	Pancreatic islets	GSE84133	17 138	20 125	14	4
Pancreas [36]	Mouse	Pancreatic islets	GSE84133	3772	14 878	13	3
PBMC_subset [37]	Human	Peripheral blood	10$\times $ Genomics	1000	32 738	3	1
Jurkat–293T [37]	Human	*In vitro* cell lines	10$\times $ Genomics	1544–1562	16 971	2	1

These datasets enable a multi-angle, including accuracy and robustness performance, and runtime evaluation of rare-cell identification:

(i) Real datasets Airway [33], Deng [34], Hrvatin [35], Intestinal Organoid [18], CRC metastases G1–3 (Metastasis tumours of colorectal cancer), Pancreas [36], and PBMC_subset constructed from PBMC68k [37] and contains 10 CD14$^{+}$ monocytes embedded within 1000 background Natural Killer (NK) cells and B cells, assess accuracy and robustness to biological heterogeneity;
(ii) Jurkat–293T [37], where Jurkat is the only rare-cell type, quantifies identification performance under extreme type imbalance;
(iii) Hrvatin and CRC metastases G1 are used to evaluate runtime via stratified down-sampling; and
(iv) CRC metastases G1 analysed as a case study.

### Baselines

We compared RAG with six state-of-the-art rare-cell identification methods covering major methodological paradigms. Feature-driven methods, including GiniClust3, which employs dual-channel gene selection; CIARA, leveraging local gene-enrichment statistics; and SCA with surprisal-based representation. Recursive clustering method scCAD, adopting iterative decomposition. Density-based FiRE using density scoring, aKNNO, a recent adaptive graph-based single-cell clustering method. All methods were run with the authors’ recommended parameters, and all datasets were preprocessed identically. All experiments were conducted on a workstation equipped with an Intel 12th Gen Core i5-12600KF CPU (10 cores, 16 threads, 3.7 GHz) and 32 GB of RAM.

### Evaluation measurements

As in scCAD [20] and SCA [13], we evaluate rare-cell identification accuracy and robustness using precision, F1 score, and rare-type coverage rate (RCR). In addition, we further introduce an identification limit (IL) to quantify methods’ robustness, and runtime to assess efficiency.

(1) The precision measures cluster purity with respect to rare cells, defined as:
  (17) \begin{align*}& \mathrm{Precision} = \frac{TP}{TP + FP}, \qquad\end{align*}
  where TP and FP are from the confusion matrix between the representative predicted cluster and the true cells. The final precision is obtained by macro-averaging over all rare types to ensure each rare population contributes equally, and higher is better.
(2) The F1 score comprehensively measures the accuracy and sensitivity of rare-cell identification, defined as:
  (18) \begin{align*}& \mathrm{F1}=\frac{2TP}{2TP+FP+FN},\end{align*}
  where TP, FP, and FN are from the confusion matrix too. Also, the F1 score is macro-averaged, and the higher the score, the better. Because different methods output either explicit rare clusters or full clusters, we select the cluster that maximizes the rare-cell F1-score to ensure comparability.
(3) The RCR measures the extent of recovery for all rare-cell types. A rare type is considered successfully identified if its representative predicted cluster satisfies: (i) the dominant annotated cell type of the predicted cluster equals the rare type, and (ii) at least $30\%$ of cells annotated as the rare type are in the predicted cluster [20, 45]. Specifically defined as:
  (19) \begin{align*}& \mathrm{RCR} = \frac{1}{|\mathcal{T}|} \sum_{t\in \mathcal{T}} \mathbb{I}\!\left( \mathrm{dom}(C_{t}^{*}) = t \;\land\; \frac{|C_{t}^{*} \cap \mathcal{X}_{t}|}{|\mathcal{X}_{t}|} \ge 0.3 \right),\end{align*}
  where $\mathcal{T}$ is the set of rare-cell types, $t$ is a rare type, $C_{t}^{*}$ is the representative predicted cluster for type $t$, and $\mathcal{X}_{t}$ is the set of cells annotated as type $t$. $\mathrm{dom}(C_{t}^{*})$ denotes the annotated cell type with the largest proportion in cluster $C_{t}^{*}$. $\mathbb{I}(\cdot )$ is the indicator function, and $\land $ denotes logical AND. Notely, the corresponding metric in scCAD is the number of identified rare types. In this paper, the results have been normalized to make them comparable across different datasets.
(4) The IL quantifies identification performance under extreme type imbalance, defined as:
  (20) \begin{align*}& IL = \min \{\, l \in \mathcal{L} \mid \mathrm{RCR}(l)=1 \,\},\end{align*}
  where $l$ is the rare-cell proportion of datasets, $\mathcal{L}$ is the set of evaluated proportions, and $\mathrm{RCR}(l)$ is computed by applying the above RCR definition. The lower the IL, the better the method performs in extreme type imbalance situations.
(5) We also use runtime to measure how efficiently a method runs. Runtime is the wall time consumed by the running process of the method. The less time-consuming the method is, the more efficient it is.

## Results

### Regularized adaptive graph has superior rare-type identification capability on real datasets

We benchmark rare-type identification methods on 10 real scRNA-seq datasets to assess the performance across diverse species, tissues, and biological contexts. RAG achieves superior overall rare-type identification accuracy and robustness.

**Figure 2:**
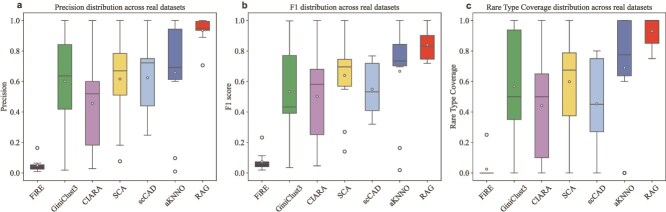
Performance comparison of various methods on real datasets. Boxplots summarize the distribution of (a) precision, (b) F1 scores, and (c) RCR across datasets.

As shown in Fig. 2, RAG overall outperforms all baselines across precision, F1 score, and RCR. The aKNNO has the strongest average performance and is, on average, the best-performing in baselines. SCA, scCAD, GiniClust3, and CIARA exhibit moderate and variable effects across datasets, whereas FiRE frequently fails to recover rare populations in multi-type settings. RAG consistently achieves the highest median precision, F1 score, and RCR across all datasets, indicating improved accuracy. In addition, RAG’s interquartile ranges are smaller than those of all baseline methods, indicating improved robustness under diverse datasets.

**Table 2:** Comparison results per dataset.

	Precision			F1			RCR		
Dataset	Best	RAG	$\boldsymbol{\Delta }$	Best	RAG	$\boldsymbol{\Delta }$	Best	RAG	$\boldsymbol{\Delta }$
Airway	**0.97**	0.89	−0.08	**0.80**	0.74	−0.06	1.00	**1.00**	+0.00
Deng	0.60	**1.00**	+0.40	0.72	**0.87**	+0.14	0.60	**1.00**	+0.40
Hrvatin	1.00	**1.00**	+0.00	**1.00**	0.97	−0.03	1.00	**1.00**	+0.00
Intestinal organoid	0.25	**0.98**	+0.73	0.32	**0.89**	+0.57	0.25	**1.00**	+0.75
CRC metastases G1	**1.00**	0.95	−0.05	**0.94**	0.90	-0.04	1.00	**1.00**	+0.00
CRC metastases G2	0.80	**0.93**	+0.13	0.75	**0.76**	+0.01	0.80	**0.80**	+0.00
CRC metastases G3	0.74	**0.95**	+0.21	0.72	**0.72**	+0.00	0.75	**0.75**	+0.00
Pancreas (human)	0.71	**0.71**	+0.00	0.72	**0.74**	+0.02	**0.75**	0.75	+0.00
Pancreas (mouse)	0.76	**0.94**	+0.18	0.66	**0.80**	+0.13	0.67	**1.00**	+0.33
PBMC_subset	1.00	**1.00**	+0.00	0.94	**1.00**	+0.06	1.00	**1.00**	+0.00
Average of best	0.78	**0.93**	+0.15 (19%)	0.76	**0.84**	+0.08 (11%)	0.78	**0.93**	+0.15 (19%)
Average of second (aKNNO)	0.66	**0.93**	+0.28 (42%)	0.66	**0.84**	+0.17 (26%)	0.69	**0.93**	+0.24 (35%)
Average of all	0.50	**0.93**	+0.43 (87%)	0.49	**0.84**	+0.35 (70%)	0.46	**0.93**	+0.47 (101%)

The method with the best performance is highlighted in bold. Compared with these per-metric best baselines, RAG achieves average improvements of 19%, 11%, and 19% in precision, F1 score, and RCR, respectively. Compared with aKNNO, the second-ranked method on average, RAG improves precision, F1 score, and RCR by 42%, 26%, and 35%, respectively. Relative to the mean performance across all methods, the improvements further increase to 87%, 70%, and 101%, indicating stronger overall accuracy. Across datasets, the maximum decreases and increases for precision, F1 score, and RCR are $-0.08$ and $+0.73$, $-0.06$ and $+0.57$, and $+0.00$ and $+0.75$, respectively, showing that RAG has small worst-case degradations and larger best-case gains across datasets. Detailed per-method results are provided in Supplementary Table S1.

### Regularized adaptive graph has the highest identification performance under extreme type imbalance

We evaluate all methods under extreme type imbalance using controlled Jurkat-293T mixtures. RAG consistently delivers the strongest rare-cell identification accuracy and robustness. On 10 controlled Jurkat-293T mixtures spanning 4–22 rare cells (0.26%–1.4%), RAG attains the highest precision and F1 score across all rare-cell counts and the lowest IL.

**Figure 3:**
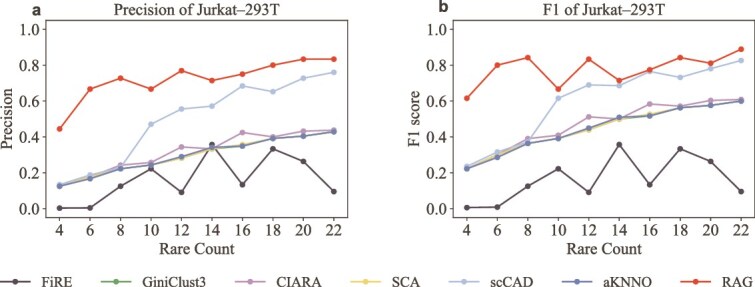
Precision and F1 score of various methods on controlled Jurkat-293T mixtures with varying rare-cell counts. The line graphs show the trends in (a) precision and (b) F1 score as a function of rare-cell counts.

Figure 3 shows that across methods, performance improves as the rare-cell count increases, because a larger population yields stronger and more separable rare-cell signals. RAG consistently achieves the highest precision and F1 score across all rare-cell counts, exceeding the second-best method, scCAD, by 0.177 (29.4%) in F1 and 0.223 (45.0%) in precision on average, demonstrating its accuracy. When the number of rare cells exceeds 6, RAG attains precision above 0.5. In contrast, the strongest competing method, scCAD, requires at least 12 rare cells to reach the same level, while the remaining methods never exceed 0.5. A similar trend is observed for the F1 score, indicating that RAG is more robust under extreme type imbalance. It is worth noting that additional misdetected rare cells can cause fluctuations in metrics, which explains why RAG does not exhibit a strictly monotonic increase with increasing rare-cell count. The same reasoning applies to other methods, and the effect is amplified when rare-cell detection is weaker, where a single missed cell can cause larger metric variations, as observed for FiRE.

We further evaluate identification under extreme type imbalance using the detection limit $IL$, with results summarized in Table 3; the best result is highlighted in bold. RAG recovers the Jurkat type at 8 rare cells, corresponding to $IL=0.52\%$, whereas scCAD requires 12 rare cells, yielding a higher detection limit of $0.77\%$. The remaining methods do not achieve successful type recovery within the tested range. Overall, RAG exhibits the strongest identification capability under extreme type imbalance. Complete numerical results for all metrics are provided in Supplementary Table S2.

**Table 3:** Detection limit ${\mathit{IL}}$ on the Jurkat–293T mixture datasets.

Method	FiRE	GiniClust3	CIARA	SCA	scCAD	aKNNO	RAG
$\boldsymbol{IL}$	–	–	–	–	0.77%	–	**0.52%**

### Stage-wise ablation and complementary effects of the regularized adaptive graph

**Figure 4:**
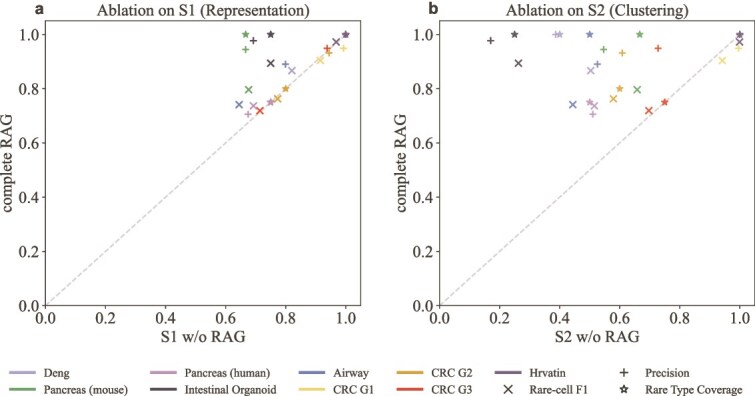
Ablation study of the RAG operator in RAG. Variants remove the RAG operator from (a) the representation learning stage and (b) the clustering stage. Colours indicate datasets, and marker shapes denote F1 score, precision, and RCR, respectively.

We conduct a stage-wise ablation study to isolate the contributions of the two uses of the RAG operator in RAG. Both uses of the RAG operator benefit RAG, with the clustering-stage use providing the dominant gains in rare-type separation, as shown in Fig. 4. Each scatter plot compares the full model with a variant in which one stage-wise use of the RAG operator is removed; points above the diagonal indicate performance gains attributable to the corresponding component.

In Fig. 4a, removing the representation-stage RAG (S1 w/o RAG) reduces rare-cell F1, precision, and RCR on several datasets. The full model almost always attains equal or higher scores, indicating that representation learning with the RAG-derived neighbourhood improves embedding quality and provides a stronger robustness for downstream clustering. Figure 4b shows a larger performance drop when the clustering-stage RAG is removed (S2 w/o RAG). Most points lie well above the diagonal, demonstrating that regularized adaptive graph construction during clustering is critical for maintaining cluster separation and preventing rare populations from being embedded into nearby dominant groups.

Additionally, some points in Fig. 4 lie close to or even below the diagonal, which can be explained from the perspective of the rare cell recognition mechanism. The RAG method focuses on rare cell identification, amplifying subtle differences among cells and highlighting rare cell features, while being prone to over-clustering. For datasets with hard-to-distinguish rare cells and low baseline Precision, F1, and RCR, amplifying cell differences yields better performance, which accounts for RAG’s outstanding results on the Deng, Pancreas, Intestinal Organoid, and Airway datasets. On CRC G2 and CRC G3, RAG shows no performance degradation, though without obvious improvement, corresponding to points closely distributed along the diagonal. When rare cells are highly distinguishable, excessive differentiation caused by RAG may degrade metrics. For the CRC G1 and Hrvatin datasets, introducing the RAG operator brings no improvement or even a slight drop in Precision and F1. Nevertheless, these metrics remain >0.9 with RCR = 1, indicating that RAG can still recover all annotated rare-cell types in these datasets. Overall, the RAG operator effectively improves recognition accuracy for datasets with indistinguishable rare cells while maintaining competitive performance on datasets with easily distinguishable rare cells.

### Regularized adaptive graph has robust near-optimal default parameters

**Figure 5:**
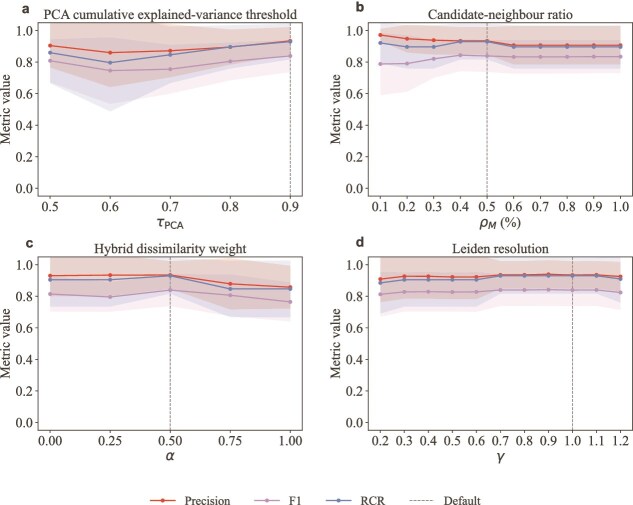
Parameter sensitivity analysis of RAG. Four key parameters were evaluated: the PCA cumulative explained-variance threshold $\tau _{\mathrm{PCA}}$, the candidate-neighbour ratio $\rho _{M}$, the hybrid dissimilarity weight $\alpha $, and the Leiden resolution parameter $\gamma $. Each point represents the mean value across 10 real scRNA-seq datasets, and shaded bands represent the standard deviation (SD) across datasets. Precision, F1 score, and RCR are shown in different colours. Vertical dashed lines indicate the selected default settings.

We conducted a systematic parameter sensitivity analysis. Four key parameters were evaluated in the main text: the PCA cumulative explained-variance threshold $\tau _{\mathrm{PCA}}$, the candidate-neighbour ratio $\rho _{M}$, the hybrid dissimilarity weight $\alpha $, and the Leiden resolution parameter $\gamma $. For each parameter setting, Precision, F1 score, and RCR were averaged across the 10 real scRNA-seq datasets, and the shaded bands indicate SD across datasets, as shown in Fig. 5.

For $\tau _{\mathrm{PCA}}$ and $\alpha $, the default values correspond to the best averaged performance. All three averaged metrics reach their highest values at $\tau _{\mathrm{PCA}}=0.9$, with Precision = 0.935, F1 score = 0.839, and RCR = 0.930. Similarly, $\alpha =0.5$ gives the best averaged Precision, F1 score, and RCR, supporting a balanced contribution of Euclidean magnitude-sensitive proximity and cosine direction-sensitive expression-pattern similarity. These results support the selected default values $\tau _{\mathrm{PCA}}=0.9$ and $\alpha =0.5$.

We next examined the candidate-neighbour ratio $\rho _{M}$ and the Leiden resolution parameter $\gamma $. For $\rho _{M}$, we tested values from 0.1% to 1.0% at a step of 0.1%. The averaged metrics changed mildly across this range, with a maximum relative variation of $\sim $7.0%. The default value $\rho _{M}=0.5\%$ achieved near-best overall performance, with RCR tied at the maximum and F1 score close to the optimum. For $\gamma $, we tested values from 0.2 to 1.2 at a step of 0.1%. The maximum relative variation of the averaged metrics was below 5.0%, indicating that Leiden clustering on the RAG graph is relatively insensitive to the resolution parameter. The default value $\gamma =1.0$ lies within a broad high-performance plateau.

Finally, to address the small-dataset edge case where $\rho _{M} N$ may produce too few candidate neighbours, we introduced a lower bound $M_{\min }$ and evaluated it in Supplementary S2. The candidate-neighbour count is defined as $M=\max (\rho _{M} N, M_{\min })$. Testing $M_{\min }$ from 3 to 7 showed that $M_{\min }=5$ achieved the highest average F1 score while maintaining near-best Precision and RCR. Together, these results indicate that RAG does not rely on narrowly tuned hyperparameters and that the selected default settings are robust or near-optimal across datasets.

### Regularized adaptive graph is faster than the most accurate method and has a nearly linear runtime

To quantify computational efficiency, we evaluated runtime as subset sizes increased across large datasets. RAG is more efficient than the most accurate aKNNO and exhibits approximately near-linear runtime growth while maintaining a stable RCR, indicating that its improved accuracy does not come at the cost of poor runtime.

**Figure 6:**
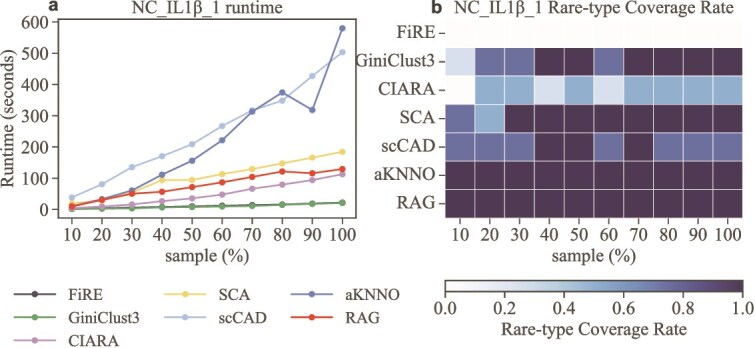
(a) Runtime and (b) RCR of methods on sampled subsets of CRC metastases G1, containing 27 201 cells, extracting a 10%–100% uniform sampling subset at 10% intervals.

As shown in Fig. 6a, scCAD becomes the most time-consuming method as the dataset size grows, consistent with its iterative cluster decomposition and merging procedure. aKNNO and SCA are also relatively time-consuming, consistent with their iterative refinement procedures. By contrast, FiRE is extremely fast because it assigns per-cell rarity scores via sketching, and GiniClust3 remains lightweight due to its feature-driven design and efficient consensus construction; however, these faster methods, including CIARA, sacrificed precision and do not match RAG in rare-type recovery on the same datasets, as detailed in Fig. 6b.

RAG runs faster than the strongest accuracy-oriented baseline, avoiding iterative approaches to improve performance and instead relying on one-pass regularized adaptive graphs for information-theoretic representation learning and clustering. We performed the same evaluation on another large dataset of 48 266 cells (Hrvatin) and obtained consistent conclusions; the detailed results are provided in the Supplementary Fig. S1.

### Regularized adaptive graph identifies rare populations in colorectal tumour tissue

Beyond clustering-level evaluation, we further examined whether the rare populations resolved by RAG were supported by gene-level biological evidence and consistent marker signatures. We first present a detailed case study on colorectal tumour tissue (CRC metastases G1).

**Figure 7:**
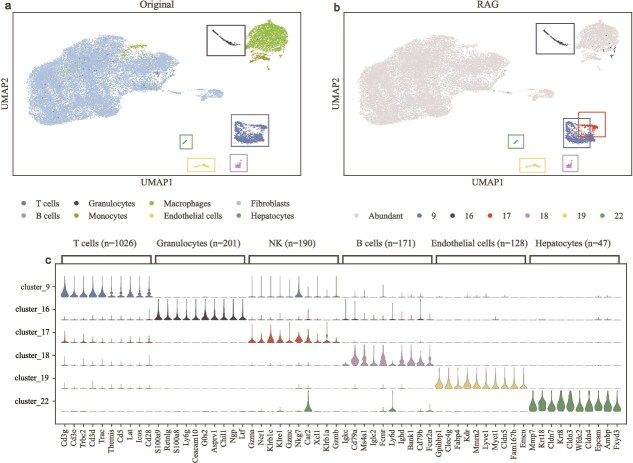
Visualization analysis of RAG’s results on the colorectal tumour. (a) UMAP 2D embedding of the cells with colour-coded original cell-type annotations. (b) UMAP 2D embedding of clusters predicted by RAG, rare and the marker-supported NK-associated candidate subcluster are marked with distinct colours and rectangles, including all annotated rare populations and an additional NK subcluster separated from T cells. (c) Violin plots showing the expression distributions of the top 10 most upregulated differentially expressed genes for each rare-cell cluster, the T and NK cell clusters. Genes belonging to the same cell cluster are indicated with the same colour.

This dataset was annotated using Louvain clustering, followed by expert curation. The composition is highly imbalanced, with dominant fibroblasts and multiple low-frequency immune/stromal populations, making it a challenging benchmark as shown in Fig. 7a. Applying RAG yields clusters with one-to-one correspondence to reference labels and additionally separates a marker-supported NK-associated subcluster ($n=190$) from T cells, as shown in Fig. 7b.

Table 4 compares rare-type recovery across methods (check marks identified as an independent cluster; cross marks failed, with the dominant misassigned lineage reported). RAG is more accurate joint recovery of annotated rare-cell populations and separation of the marker-supported NK-associated subcluster from T cells than the other evaluated methods, whereas baselines often merge rare types into major clusters or misassign them; baseline UMAPs are shown in Supplementary Figs S3–S8.

**Table 4:** Comparison of different methods on the CRC metastases G1 dataset in identifying rare-cell populations.

	RAG	aKNNO	SCA	scCAD	CIARA	GiniClust3	FiRE
Hepatocytes	✓;precision = **1.0**	✓;precision = 1.0	✓	$\times $	$\times $ ; in endothelial cells	✓	$\times $
Endothelial cells	✓;precision = **0.99**	✓;precision = 0.83	✓	$\times $	$\times $ ; in hepatocytes	✓	$\times $
Granulocytes	✓;precision = **0.82**	✓;precision = 0.65	✓	✓	✓	✓	$\times $
B cells	✓;precision = **0.98**	✓;precision = 0.96	✓	✓	$\times $ ; in monocytes	✓	$\times $
NK	✓	✓	$\times $ ; in T cells	✓	$\times $ ; in T cells	$\times $ ; in T cells	$\times $

Differential expression analysis based on Wilcoxon rank-sum further supports these interpretations; the resolved candidate NK-associated subcluster shows strong expression of canonical NK markers (e.g. Ncr1, Klrb1a, Klrd1, Gzma, and Gzmc) curated in CellMarker 2.0 [46, 47], and other rare clusters display consistent marker signatures in Fig. 7c, with a more detailed differential expression analysis description and results in Supplementary S6 and source code.

Because the NK-associated signal appears only in CRC G1, we further evaluated RAG under a multi-sample setting by combining CRC G1 and CRC G3 into a three-batch dataset and applying Harmony integration. In this pooled Harmony-integrated analysis, the NK-associated signal was attenuated and was not resolved as an independent cluster. This result reveals a practical limitation of applying RAG in a global multi-sample setting: rare populations restricted to one sample may be weakened by the pooled background. Although RAG is technically compatible with standard batch-correction tools such as Harmony, this compatibility may not remove the risk that highly sample-restricted rare signals may be diluted after pooled analysis or batch correction. Therefore, for multi-subject datasets, we recommend sample-specific RAG analysis and marker-gene validation. The corresponding results are provided in Supplementary Fig. S9.

### Cross-dataset validation of regularized adaptive graph-resolved small clusters

To further assess whether RAG-resolved small clusters correspond to known annotated populations, marker-supported subpopulations, or potential over-clustering artefacts, we extended the analysis to three additional benchmark datasets from distinct tissue contexts, including airway epithelium, mouse pancreas, and human pancreas. In each dataset, RAG-resolved small clusters were interpreted using the same candidate-screening and marker-validation workflow.

To systematically interpret RAG-resolved small clusters, we prioritized them using a workflow based on cluster frequency and marker distinctiveness [48]. Clusters accounting for 0.1%–3% of all cells were retained as candidate small clusters and analysed by one-versus-rest Wilcoxon differential expression using the log-normalized full-gene expression matrix. For each candidate cluster $C_{i}$, a marker set $G_{i}$ was defined using FDR $\leq 0.05$, $\log _{2}\mathrm{FC} \geq 2$, and $\Delta \mathrm{pct} \geq 0.2$, and ranked by 


(21) \begin{align*}& U_{i}=\sum_{j\neq i}|G_{i}\setminus G_{j}|,\qquad{\mathrm{RPS}}_{i}=\min\left(\frac{|G_{i}|}{10},1\right)\times U_{i}.\end{align*}

Cluster size was used only for candidate screening, whereas RPS$_{i}$ prioritized clusters with sufficiently large and distinctive marker sets. Ranked candidates were then classified as known annotated populations, additional marker-supported subpopulations, or low-confidence/over-segmented clusters based on top markers, dominant original annotations, and literature-supported evidence.

**Figure 8:**
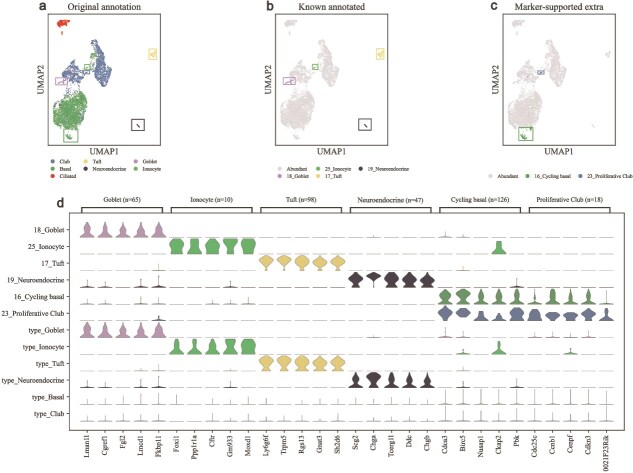
Visualization analysis of RAG-resolved small clusters in the airway epithelial dataset. (a) UMAP 2D embedding of cells with colour-coded original cell-type annotations. (b) UMAP 2D embedding highlighting RAG-resolved known annotated low-frequency epithelial populations, including goblet cells, ionocytes, tuft cells, and pulmonary neuroendocrine cells. (c) UMAP 2D embedding highlighting RAG-resolved marker-supported proliferative epithelial substates, including cycling basal cells and proliferative club cells. (d) Violin plots showing the expression distributions of the top 5 most upregulated differentially expressed genes for each RAG-resolved known annotated population and marker-supported substate. Genes belonging to the same cluster are indicated with the same colour.

In the airway epithelial dataset, RAG recovered multiple annotated low-frequency epithelial populations as independent small clusters, including goblet cells, ionocytes, tuft cells, and pulmonary neuroendocrine cells, consistent with published airway epithelial atlases [33, 49], as shown in Fig. 8a and b. These recovered populations were further supported by canonical marker signatures, including Dmbt1/Tff1 for goblet cells, Foxi1/Cftr for ionocytes, Trpm5/Gnat3 for tuft cells, and Chga/Snap25 for pulmonary neuroendocrine cells. RAG also separated marker-supported proliferative epithelial substates from major airway annotations, as shown in Fig. 8a and c. Within the original basal-cell annotation, RAG identified a cycling basal-cell state. Cluster 16 retained a basal-lineage background supported by basal markers such as Krt5, Aqp3, Sfn, Perp, and Bcam, and showed a strong cell-cycle programme marked by Mki67, Top2a, Ccnb1, Ccna2, Cdk1, Ube2c, Birc5, and Tpx2. This pattern is consistent with the cycling basal-cell population and injury-associated Krt5-positive basal cells in cycle reported in airway epithelial single-cell atlases [33, 49]. Within the original club-cell annotation, RAG identified a small putative proliferative club-cell state. Cluster 23 retained a club/secretory-cell background supported by markers such as Cyp2a5, Ltf, Pigr, Sftpd, Cxcl17, and Aldh1a1, and was enriched for mitotic genes, including Cdc25c, Ccnb1, Cenpf, Birc5, Ccnb2, Cdc20, Mki67, and Top2a. This pattern suggests a possible proliferative state within the club-cell compartment and is consistent with club-cell heterogeneity and epithelial-state dynamics reported in airway single-cell atlases [33, 49]. Together, these results indicate that RAG can recover annotated rare epithelial populations and reveal candidate biologically interpretable substates within major airway epithelial annotations. The top 5 most upregulated differentially expressed genes for each small cluster are shown in Fig. 8d. In both mouse and human pancreas datasets, RAG similarly recovered known rare or nonendocrine pancreatic populations and resolved marker-supported substructures within major pancreatic annotations, including ductal, endothelial, acinar, and beta-cell-associated states. The corresponding detailed small-cluster analyses are provided in Supplementary S5. The UMAP plots, marker-gene expression patterns are provided in Supplementary Figs S10–S12 for mouse pancreas, and Supplementary Figs S13–S15 for human pancreas.

After analysing the recovered annotated populations and the additional marker-supported subpopulations in the three datasets, we further examined low-confidence or over-segmented small clusters. We divided all RAG-derived small clusters into three groups: known annotated populations, additional marker-supported subpopulations, and low-confidence or over-segmented clusters. For each dataset, the low-confidence proportion was calculated as the number of low-confidence or over-segmented clusters divided by the total number of candidate small clusters. The proportions were 45.45%, 57.14%, and 50.00% for the airway epithelial, mouse pancreas, and human pancreas datasets, respectively, with an average of 50.86% (Table 5). Although low-confidence clusters were present in all datasets, a substantial proportion of RAG-derived small clusters matched known annotated populations or marker-supported subpopulations. This indicates that RAG, combined with downstream differential expression analysis, can recover biologically meaningful rare or low-frequency cell populations and within-annotation structure.

**Table 5:** Summary of RAG-derived candidate small clusters across the three case-study datasets.

Dataset	Total candidates	Known annotated	Marker-supported extra	Low-conf./over-seg.	Low-conf. prop. (%)
Airway	11	4	2	5	45.45
Pancreas (mouse)	35	8	7	20	57.14
Pancreas (human)	28	6	8	14	50.00
Average	24.67	6.00	5.67	13.00	50.86

## Discussion

Existing rare-cell identification methods fall into three categories: feature-driven [13–17], recursive clustering [18–21], and density-based [22–24]. Most use fixed-size neighbourhood graphs, which perform well in specific scenarios but suffer from low accuracy and robustness when cell densities are nonuniform in the representation space, often misclustering rare cells. Recursive clustering handles weak signals but is computationally expensive.

We present RAG, an accurate, robust, near-linear-time approach for rare-cell identification. Its core is the regularized adaptive graph with cell-specific radius-controlled adjacency and locally scaled affinities. RAG first learns cleaner Wilcoxon-based information-theoretic representations, then reconstructs a refined graph for Leiden clustering, reducing rare-cell misembedding into major clusters. Experiments on scRNA-seq datasets show RAG overall outperforms state-of-the-art methods.

Specifically, the regularized adaptive graph controls adjacency using a cell-specific radius in the hybrid dissimilarity space and normalizes affinity magnitudes using local scales. Unlike fixed-size neighbourhood graphs, it determines retained neighbours by cell-specific radii rather than by a fixed neighbourhood size; unlike a pure adaptive bandwidth kernel, the local scale affects both adjacency and affinity; unlike standard SNN graphs, it uses shared-neighbour information as a *post-hoc* bridge-pruning step to improve graph robustness, rather than as the primary definition of edge weights.

We benchmark RAG on mixture and real datasets. Compared with six state-of-the-art methods, RAG achieves the highest precision on controlled mixtures and 10 scRNA-seq datasets, with stronger robustness in cross-dataset evaluation, and is faster than the most accurate baseline, with near-linear runtime as data increases. RAG can more accurately recover annotated rare-cell populations and resolve biologically meaningful small clusters in colorectal tumour tissue, mouse airway epithelium, and two pancreas datasets.

In addition, RAG currently uses the SCA-derived Wilcoxon-based surprisal representation to model local–global expression deviations. Although this representation provides a stable basis for the proposed graph-construction framework, alternative local-deviation scores, such as z-score or MAD-score variants, may also be integrated into the same RAG workflow. Systematically evaluating these alternative representation scores is an important direction for future work. RAG also employs adaptive regularization that may discard the boundary cells in certain geometries; future work will investigate boundary-aware adjacency control. Overall, RAG offers an accurate, robust solution for rare cell identification with promising applications in disease and developmental studies.

**Key points**

- RAG is a regularized adaptive graph-based method for rare-cell identification, whose core contribution is a regularized adaptive graph operator applied to both information-theoretic representation learning and Leiden clustering to improve rare-population preservation.
- On 10 widely used real scRNA-seq benchmarks, RAG improves over the second-ranked baseline by 42% in precision, 26% in F1 score, and 35% in RCR on average.
- Analyses of colorectal tumour tissue, mouse airway epithelium, and two pancreas datasets show that RAG can recover annotated rare-cell populations and resolve biologically meaningful small clusters with marker support.

## Supplementary Material

RAG_revised_supplementary_bbag379

Supplementary_Table_S1_bbag379

Supplementary_Table_S2_bbag379

## Data Availability

The scRNA-seq datasets are available from the NCBI GEO database or the 10$\times $ website, and the access IDs are listed in Table 1. The CRC metastases were provided by Jiangsu Cancer Hospital with permission. Data will be shared on request to the corresponding author with permission from Jiangsu Cancer Hospital. The source code is available at https://github.com/wangxingsu/RAG.

## References

[1] Bi C, Bai K, Zhang X. HiCat: a semi-supervised approach for cell type annotation. *Brief Bioinform* 2025;26:bbaf428. 10.1093/bib/bbaf428 40833274 PMC12365967

[2] Woo H, Eyun S-i. Applications and techniques of single-cell RNA sequencing across diverse species. *Brief Bioinform* 2025;26:bbaf354. 10.1093/bib/bbaf354 40698863 PMC12284766

[3] Yatim KM, Lakkis FG. A brief journey through the immune system. *Clin J Am Soc Nephrol* 2015;10:1274–81. 10.2215/CJN.10031014 25845377 PMC4491295

[4] Webb LM, Wojno EDT The role of rare innate immune cells in type 2 immune activation against parasitic helminths. *Parasitology* 2017;144:1288–301. 10.1017/S0031182017000488 28583216 PMC5962964

[5] Ebinger S, Özdemir EZ, Ziegenhain C et al. Characterization of rare, dormant, and therapy-resistant cells in acute lymphoblastic leukemia. *Cancer Cell* 2016;30:849–62. 10.1016/j.ccell.2016.11.002 27916615 PMC5156313

[6] Oren Y . Hunting down rare drug-tolerant cycling cells with watermelon. *Nat Rev Cancer* 2022;22:434–5. 10.1038/s41568-022-00483-0 35538369

[7] Carrelha J, Mazzi S, Winroth A et al. Alternative platelet differentiation pathways initiated by nonhierarchically related hematopoietic stem cells. *Nat Immunol* 2024;25:1007–19. 10.1038/s41590-024-01845-6 38816617 PMC11147777

[8] Chu X, Tian W, Ning J et al. Cancer stem cells: advances in knowledge and implications for cancer therapy. *Signal Transduct Target Ther* 2024;9:170. 10.1038/s41392-024-01851-y 38965243 PMC11224386

[9] Aissa AF, Islam ABMMK, Ariss MM et al. Single-cell transcriptional changes associated with drug tolerance and response to combination therapies in cancer. *Nat Commun* 2021;12:1628. 10.1038/s41467-021-21884-z 33712615 PMC7955121

[10] He S, Fan J, Tianwei Y. G3DC: a gene-graph-guided selective deep clustering method for single cell RNA-seq data. *Big Data Min Anal* 2024;7:809–27. 10.26599/BDMA.2024.9020011

[11] Zheng R, He Y, Huang J et al. A flexible data-driven framework for correcting coarsely annotated scRNA-seq data. *Big Data Min Anal* 2025;8:997–1010. 10.26599/BDMA.2025.9020009

[12] Liu J, Zeng W, Kan S et al. CAKE: a flexible self-supervised framework for enhancing cell visualization, clustering and rare cell identification. *Brief Bioinform* 2023;25:bbad475. 38145950 10.1093/bib/bbad475 PMC10749894

[13] DeMeo B, Berger B. SCA: recovering single-cell heterogeneity through information-based dimensionality reduction. *Genome Biol* 2023;24:195. 10.1186/s13059-023-02998-7 37626411 PMC10464206

[14] Jiang L, Chen H, Pinello L et al. GiniClust: detecting rare cell types from single-cell gene expression data with Gini index. *Genome Biol* 2016;17:144. 10.1186/s13059-016-1010-4 27368803 PMC4930624

[15] Tsoucas D, Yuan G-C. GiniClust2: a cluster-aware, weighted ensemble clustering method for cell-type detection. *Genome Biol* 2018;19:58. 10.1186/s13059-018-1431-3 29747686 PMC5946416

[16] Dong R, Yuan G-C. GiniClust3: a fast and memory-efficient tool for rare cell type identification. *BMC Bioinformatics* 2020;21:158. 10.1186/s12859-020-3482-1 32334526 PMC7183612

[17] Lubatti G, Stock M, Iturbide A et al. CIARA: a Acluster-independent algorithm for identifying markers of rare cell types from single-cell sequencing data. *Development* 2023;150:dev201264. 10.1242/dev.201264 37294170

[18] Grün D, Lyubimova A, Kester L et al. Single-cell messenger RNA sequencing reveals rare intestinal cell types. *Nature* 2015;525:251–5. 10.1038/nature14966 26287467

[19] Herman JS, Sagar, Gruen D. Fateid infers cell fate bias in multipotent progenitors from single-cell RNA-seq data. *Nat Methods* 2018;15:379–86. 10.1038/nmeth.4662 29630061

[20] Yunpei X, Wang S, Feng Q et al. ScCAD: cluster decomposition-based anomaly detection for rare cell identification in single-cell expression data. *Nat Commun* 2024;15:7561. 39215003 10.1038/s41467-024-51891-9 PMC11364754

[21] Wegmann R, Neri M, Schuierer S et al. CellSIUS provides sensitive and specific detection of rare cell populations from complex single-cell RNA-seq data. *Genome Biol* 2019;20:142. 10.1186/s13059-019-1739-7 31315641 PMC6637521

[22] Jindal A, Gupta P, Jayadeva et al. Discovery of rare cells from voluminous single cell expression data. *Nat Commun* 2018;9:4719. 10.1038/s41467-018-07234-6 30413715 PMC6226447

[23] Fa B, Wei T, Zhou Y et al. GapClust is a light-weight approach distinguishing rare cells from voluminous single cell expression profiles. *Nat Commun* 2021;12:4197. 10.1038/s41467-021-24489-8 34234139 PMC8263561

[24] Sun X, Liu Y, An L. Ensemble dimensionality reduction and feature gene extraction for single-cell RNA-seq data. *Nat Commun* 2020;11:5853. 10.1038/s41467-020-19465-7 33203837 PMC7673125

[25] Traag VA, Waltman L, Van Eck NJ. From Louvain to Leiden: guaranteeing well-connected communities. *Sci Rep* 2019;9:1–12. 30914743 10.1038/s41598-019-41695-z PMC6435756

[26] Ding J, Regev A. Deep generative model embedding of single-cell RNA-seq profiles on hyperspheres and hyperbolic spaces. *Nat Commun* 2021;12:2554. 10.1038/s41467-021-22851-4 33953202 PMC8099904

[27] Kim T, Chen IR, Lin Y et al. Impact of similarity metrics on single-cell RNA-seq data clustering. *Brief Bioinform* 2019;20:2316–26. 10.1093/bib/bby076 30137247

[28] Zelnik-Manor L, Perona P. Self-tuning spectral clustering. *Adv Neural Inf Proces Syst* 2004;17:1601–8.

[29] Hao Y, Stuart T, Kowalski MH et al. Dictionary learning for integrative, multimodal and scalable single-cell analysis. *Nat Biotechnol* 2024;42:293–304. 10.1038/s41587-023-01767-y 37231261 PMC10928517

[30] Alexander Wolf F, Angerer P, Theis FJ. SCANPY: large-scale single-cell gene expression data analysis. *Genome Biol* 2018;19:15. 10.1186/s13059-017-1382-0 29409532 PMC5802054

[31] Luecken MD, Theis FJ. Current Best Practices in Single-Cell Rna-Seq Analysis: A Tutorial. Molecular Systems Biology, Vol. 15. Heidelberg, Germany: EMBO Press, 2019, MSB188746. 10.15252/msb.20188746 PMC6582955 31217225

[32] Halko N, Martinsson P-G, Tropp JA. Finding structure with randomness: probabilistic algorithms for constructing approximate matrix decompositions. *SIAM Rev* 2011;53:217–88. 10.1137/090771806

[33] Montoro DT, Haber AL, Biton M et al. A revised airway epithelial hierarchy includes CFTR-expressing ionocytes. *Nature* 2018;560:319–24. 10.1038/s41586-018-0393-7 30069044 PMC6295155

[34] Deng Q, Ramsköld D, Reinius B et al. Single-cell RNA-seq reveals dynamic, random monoallelic gene expression in mammalian cells. *Science* 2014;343:193–6. 10.1126/science.1245316 24408435

[35] Hrvatin S, Hochbaum DR, Aurel Nagy M et al. Single-cell analysis of experience-dependent transcriptomic states in the mouse visual cortex. *Nat Neurosci* 2018;21:120–9. 10.1038/s41593-017-0029-5 29230054 PMC5742025

[36] Baron M, Veres A, Wolock SL et al. A single-cell transcriptomic map of the human and mouse pancreas reveals inter-and intra-cell population structure. *Cell Syst* 2016;3:346–360.e4. 10.1016/j.cels.2016.08.011 27667365 PMC5228327

[37] Zheng GXY, Terry JM, Belgrader P et al. Massively parallel digital transcriptional profiling of single cells. *Nat Commun* 2017;8:14049. 10.1038/ncomms14049 28091601 PMC5241818

[38] Gerniers A, Nijssen S, Dupont P. scCross: efficient search for rare subpopulations across multiple single-cell samples. *Bioinformatics* 2024;40:btae371. 10.1093/bioinformatics/btae371 38889273 PMC11256925

[39] Leary JR, Yi X, Morrison AB et al. Sub-Cluster Identification through Semi-Supervised Optimization of Rare-Cell Silhouettes (SCISSORS) in single-cell RNA-sequencing. *Bioinformatics* 2023;39:btad449. 10.1093/bioinformatics/btad449 37498558 PMC10412410

[40] Li J, Shyr Y, Liu Q. aKNNO: single-cell and spatial transcriptomics clustering with an optimized adaptive k-nearest neighbor graph. *Genome Biol* 2024;25:203. 39090647 10.1186/s13059-024-03339-y PMC11293182

[41] Wang S, Li H, Liu Y et al. Connectivity network feature sharing in single-cell RNA sequencing data identifies rare cells. *J Chem Inf Model* 2024a;64:6596–609. 10.1021/acs.jcim.4c00796 39096508

[42] Gerniers A, Bricard O, Dupont P. MicroCellClust: mining rare and highly specific subpopulations from single-cell expression data. *Bioinformatics* 2021;37:3220–7. 10.1093/bioinformatics/btab239 33830183 PMC8504615

[43] Wang S, Li H, Zhang K et al. scSID: a lightweight algorithm for identifying rare cell types by capturing differential expression from single-cell sequencing data. *Comput Struct Biotechnol J* 2024b;23:589–600. 10.1016/j.csbj.2023.12.043 38274993 PMC10809081

[44] Mallick K, Chakraborty S, Mallik S et al. A scalable unsupervised learning of scRNAseq data detects rare cells through integration of structure-preserving embedding, clustering and outlier detection. *Brief Bioinform* 2023;24:bbad125. 37185897 10.1093/bib/bbad125

[45] Yang F, Wang W, Wang F et al. scBERT as a large-scale pretrained deep language model for cell type annotation of single-cell RNA-seq data. *Nat Mach Intell* 2022;4:852–66. 10.1038/s42256-022-00534-z

[46] Chen S, Zhu H, Jounaidi Y. Comprehensive snapshots of natural killer cells functions, signaling, molecular mechanisms and clinical utilization. *Signal Transduct Target Ther* 2024;9:302. 10.1038/s41392-024-02005-w 39511139 PMC11544004

[47] Congxue H, Li T, Yingqi X et al. CellMarker 2.0: an updated database of manually curated cell markers in human/mouse and web tools based on scRNA-seq data. *Nucleic Acids Res* 2023;51:D870–6. 36300619 10.1093/nar/gkac947 PMC9825416

[48] Stuart T, Butler A, Hoffman P et al. Comprehensive integration of single-cell data cell. Cell 2019;177:1888–1902.e21. 10.1016/j.cell.2019.05.031 31178118 PMC6687398

[49] Plasschaert LW, Žilionis R, Choo-Wing R et al. A single-cell atlas of the airway epithelium reveals the CCFTR-rich pulmonary ionocyte. *Nature* 2018;560:377–81. 10.1038/s41586-018-0394-6 30069046 PMC6108322

