# Supplementary material for: RAG: a regularized adaptive graph-based method for rare-cell identification from single-cell expression data
Source: Brief Bioinform. 2026 Jul 17;27(4):bbag379. doi: 10.1093/bib/bbag379 (PMC13379077; doi:10.1093/bib/bbag379)
Supplement: RAG_revised_supplementary_bbag379 [file rag_revised_supplementary_bbag379.pdf]

Supplementary

S1 Runtime Evaluation on Hrvatin

RAG is faster than the accuracy-oriented baselines and has near-linear runtime on Hrvatin.

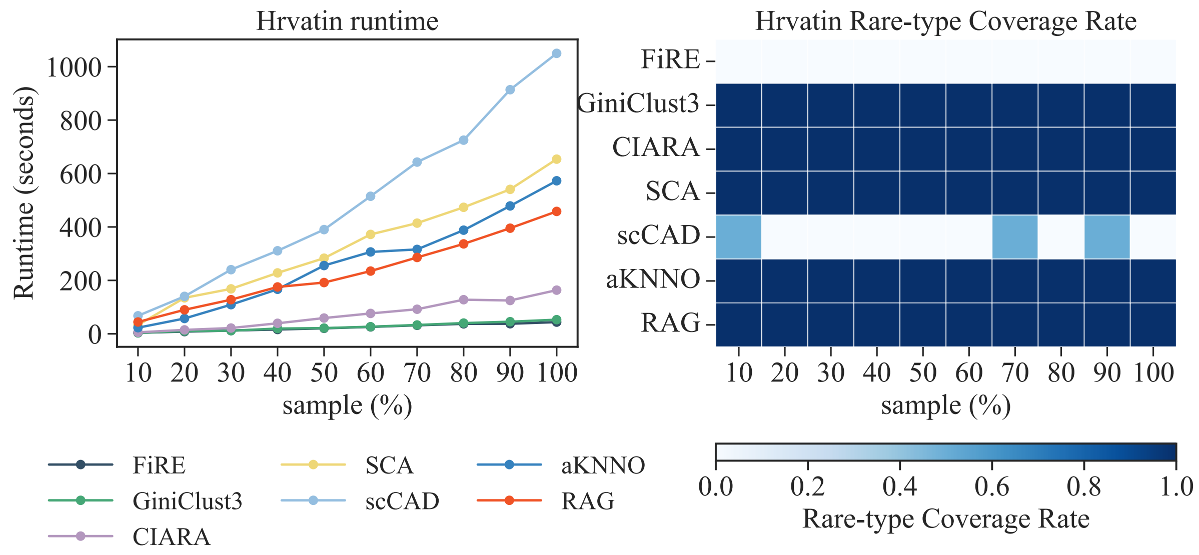

Figure S1: **a** Runtime and **b** rare-type coverage rate (RCR) of methods on sampled subsets of Hrvatin, containing 48,266 cells, with 10%–100% uniform sampling at 10% intervals.

Alt text: Two-panel plot showing runtime and rare-type coverage rate as the sampled subset size increases on the Hrvatin dataset. The panels compare RAG with baseline methods and show that RAG maintains stable RCR while exhibiting near-linear runtime growth.

S2 Sensitivity Analysis of Candidate-Count Lower Bound

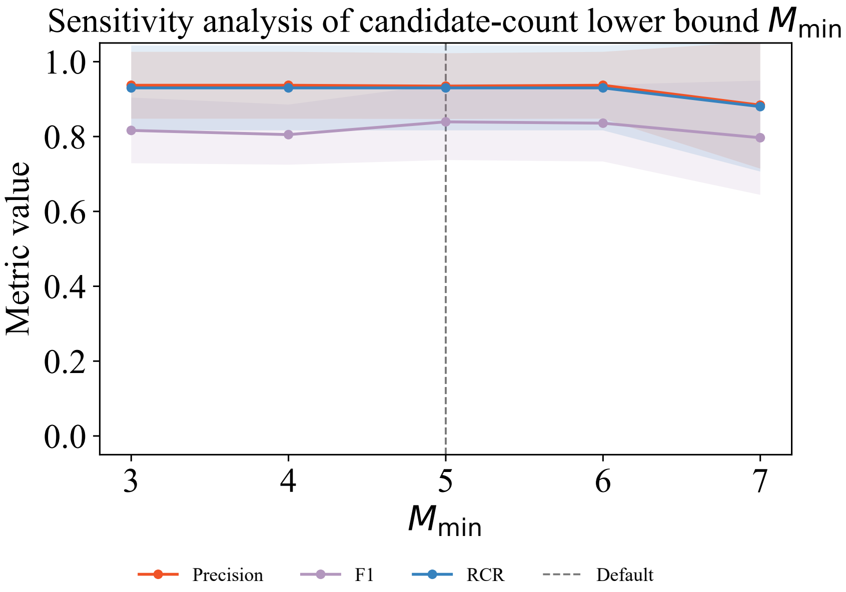

Figure S2: Sensitivity analysis of the candidate-count lower bound $M_{\min}$. Precision, F1 score, and rare-type coverage rate (RCR) are averaged across the ten real scRNA-seq datasets. The shaded bands represent the standard deviation (SD) across datasets. The vertical dashed line indicates the default setting $M_{\min} = 5$.

Alt text: Line plot showing how Precision, F1 score, and rare-type coverage rate change when the lower bound $M_{\min}$ varies from 3 to 7. Precision and RCR remain nearly stable from $M_{\min} = 3$ to 6, while F1 score reaches its highest value at $M_{\min} = 5$. All three metrics decrease when $M_{\min} = 7$.

We further examined the lower bound $M_{\min}$ used in the candidate-neighbour count definition $M = \max(\rho_M N, M_{\min})$. This lower bound prevents the candidate pool from becoming too small on small datasets, where a purely ratio-based setting may provide too few candidate neighbours for stable local-structure estimation.

When $M_{\min}$ varies from 3 to 6, RAG maintains stable Precision and RCR, with Precision around 0.935–0.937 and RCR fixed at 0.930. This indicates that this range provides a sufficient minimum candidate pool for small datasets without excessively expanding the local candidate neighbourhood. The default setting $M_{\min} = 5$ achieves the highest average F1 score of 0.839, while $M_{\min} = 6$ yields a very similar F1 score of 0.836. When $M_{\min}$ is further increased to 7, Precision, F1 score, and RCR decrease to 0.884, 0.797, and 0.880, respectively, suggesting that an overly large lower bound may introduce excessive candidates for small datasets and weaken local adjacency control. These results support $M_{\min} = 5$ as a stable default lower bound, because it maximises average F1 score while maintaining near-best Precision and RCR.

S3 Baseline UMAPs on CRC Metastases G1

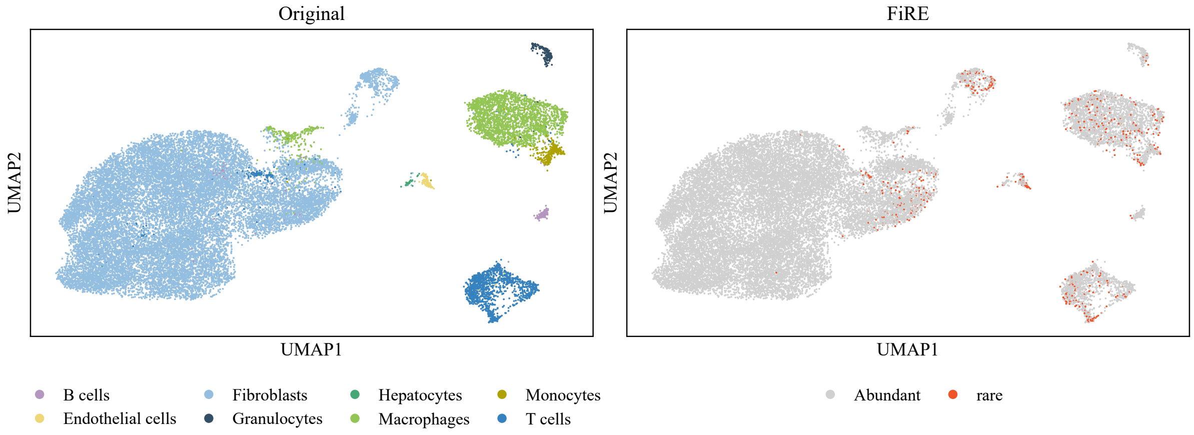

Figure S3: FiRE. UMAP visualisation produced by FiRE on the CRC metastases G1 dataset.

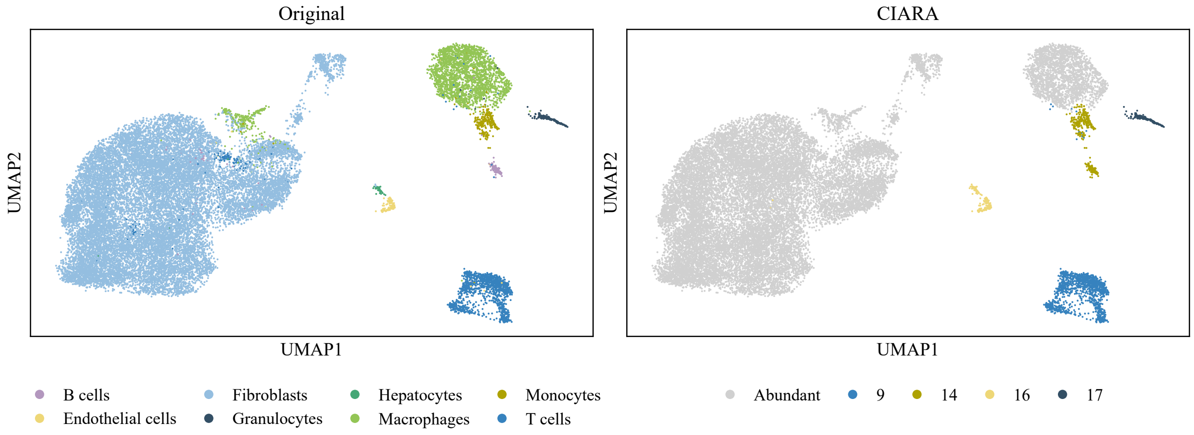

Figure S4: CIARA. UMAP visualisation produced by CIARA on the CRC metastases G1 dataset.

Alt text for Supplementary Figs. S3–S8: UMAP panels comparing the reference annotation with baseline method outputs on CRC metastases G1. These figures show how rare and major populations are separated or merged across the evaluated baseline methods.

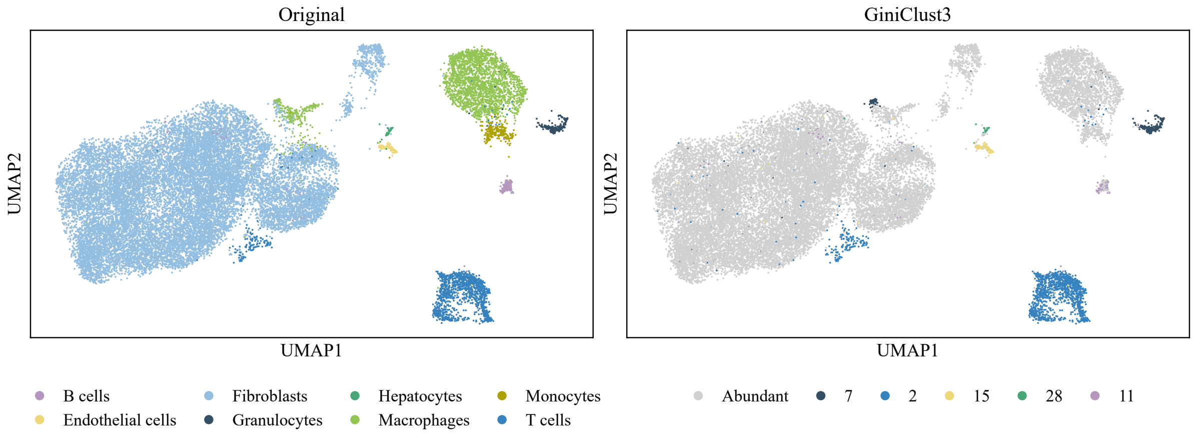

Figure S5: GiniClust3. UMAP visualisation produced by GiniClust3 on the CRC metastases G1 dataset.

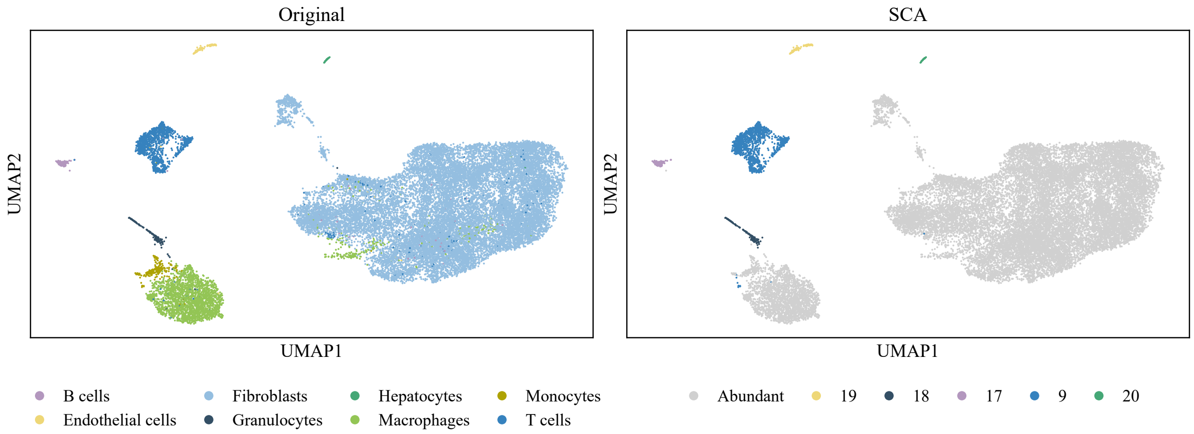

Figure S6: SCA. UMAP visualisation produced by SCA on the CRC metastases G1 dataset.

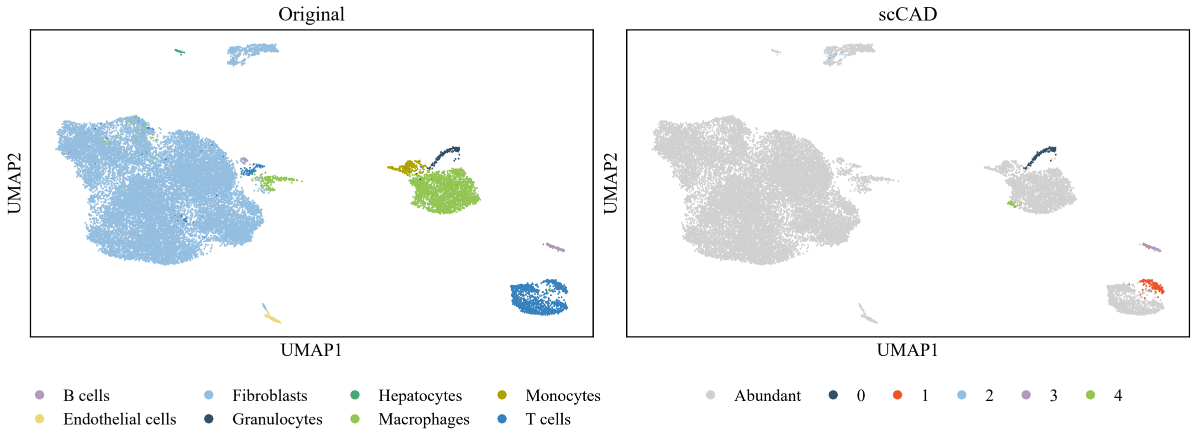

Figure S7: scCAD. UMAP visualisation produced by scCAD on the CRC metastases G1 dataset.

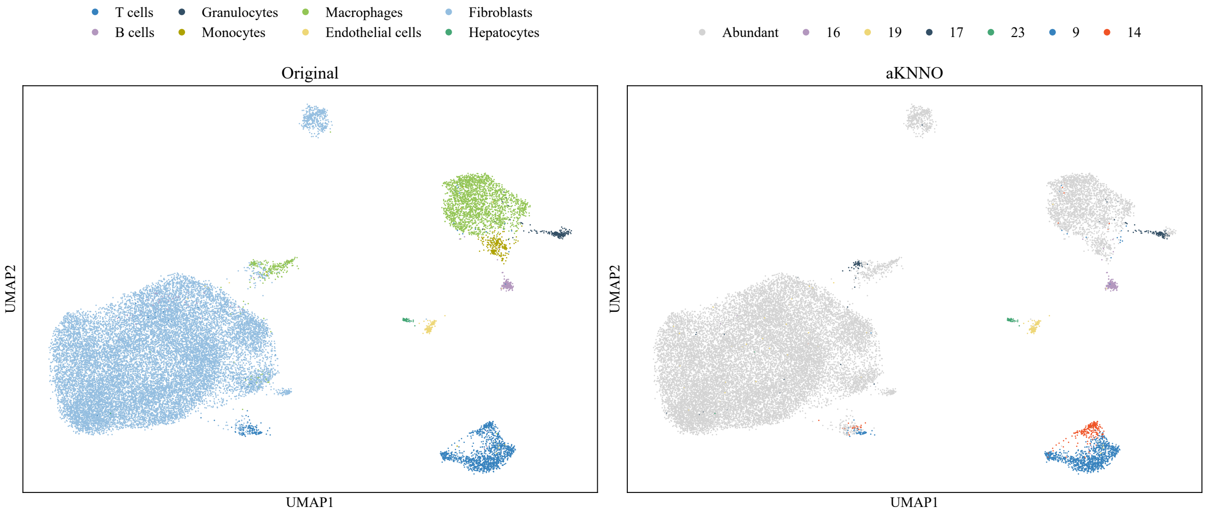

Figure S8: aKNNO. UMAP visualisation produced by aKNNO on the CRC metastases G1 dataset.

S4 Multi-Sample Analysis with Harmony Integration

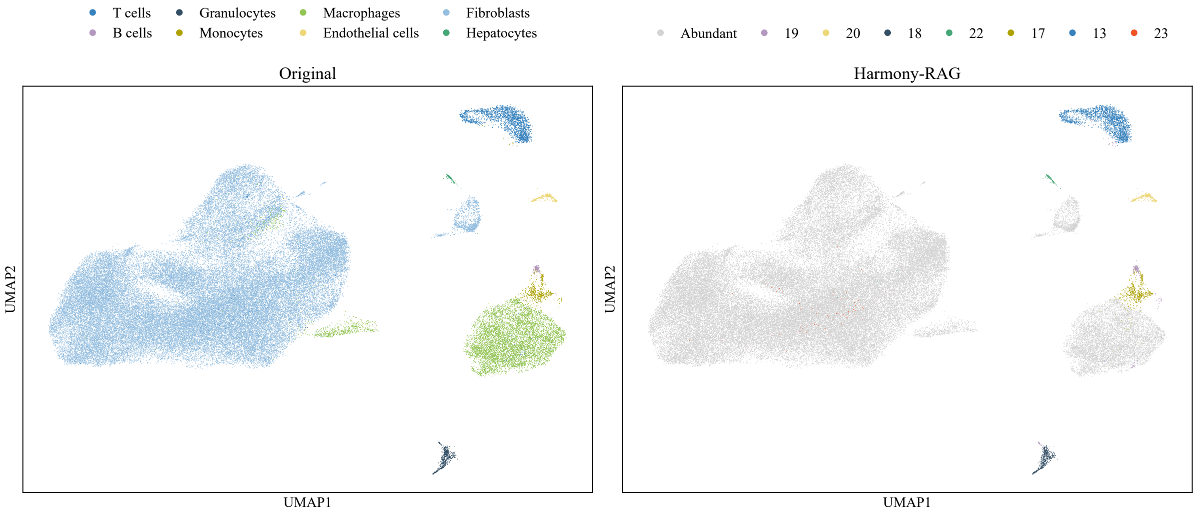

Figure S9: Multi-sample analysis of RAG after Harmony integration. CRC metastases G1 and G3 were pooled into a three-batch dataset to evaluate whether a sample-restricted rare population can be recovered after batch correction. RAG was applied to the Harmony-integrated representation of the pooled data. The NK-associated signal, which appears only in CRC metastases G1, was not resolved as an independent cluster, indicating that sample-restricted rare populations may be diluted in pooled analysis even after Harmony integration and require sample-aware validation.

Alt text: UMAP panels showing RAG results on the pooled CRC metastases G1 and G3 dataset after Harmony integration. The NK-associated signal is not resolved into an independent cluster, illustrating the difficulty of recovering sample-restricted rare populations in pooled multi-sample analyses after batch correction.

S5 Cross-dataset validation of RAG-resolved small clusters

Airway

Low-confidence and over-segmented clusters. In the GSE103354 airway epithelial dataset, five small clusters were not retained as marker-supported biological populations. Clusters 20, 21, and 22 were all derived from the original Ciliated annotation and retained ciliated-cell identity without a coherent additional marker programme; they were therefore treated as Ciliated annotation over-segmentation. Clusters 14 and 15 were derived from the original Club annotation. Although they showed secretory Club-like marker differences, these clusters largely overlapped with the original Club programme and were therefore interpreted as low-confidence data-driven Club splits rather than established independent epithelial populations. Thus, these five clusters were excluded from the marker-supported biological subpopulation analysis.

Mouse pancreas

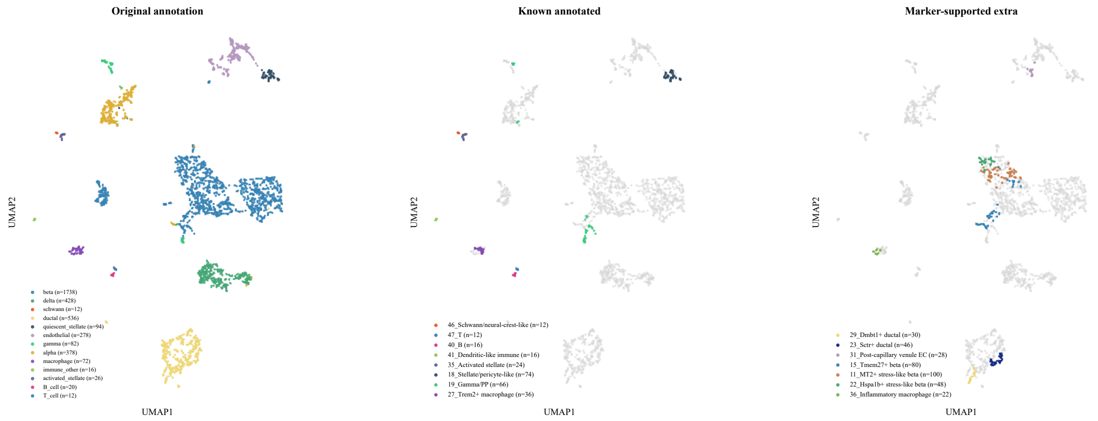

Figure S10: UMAP visualisation of RAG-derived marker-supported small clusters in the mouse pancreas dataset.

Alt text: UMAP panels showing original annotations, known annotated small clusters, and marker-supported extra clusters in the mouse pancreas dataset.

Mouse pancreas dataset: known annotated small clusters. In the mouse pancreas dataset, RAG recovered multiple known rare or non-endocrine populations as independent clusters, including Schwann cells, T cells, B cells, macrophages, dendritic-like immune cells, stellate/pericyte-like cells, activated stellate cells, and gamma/PP cells, consistent with published mouse pancreas single-cell atlases and curated marker resources [Baron et al., 2016, Hu et al., 2023]. These clusters showed canonical marker signatures, including Sox10/Ngfr for Schwann cells, Cd3d/Trac for T cells, Ms4a1/Cd79b for B cells, Lyz2/C1qb for macrophages, Ccr7/Flt3 for dendritic-like immune cells, Dcn/Col1a1/Lum for activated stellate cells, Pdgfrb/Rgs5 for stellate/pericyte-like cells, and Ppy/Pyy for gamma/PP cells. The corresponding UMAP overview and known-cluster violin plot are provided in Supplementary Figs. S10 and S11.

Mouse pancreas dataset: marker-supported small clusters. RAG also separated marker-supported subpopulations from neighbouring major annotations.

In the ductal compartment, RAG resolved two marker-supported ductal epithelial states. Cluster 29 was marked by Dmbt1/Gabrp together with Ly6d, Krt19, Muc1, Muc20, Wfdc2, S100a14, and Fgfr2, whereas cluster 23 showed a Sctr/Cftr/Sox9-associated ductal programme with additional epithelial and activated-state markers, including Krt17, Cldn4, Lcn2, Cd44, and Hes1. These patterns are consistent with the mouse ductal substructure described in GSE84133 and with later evidence for Dmbt1/Ly6d- and Cftr-associated pancreatic ductal heterogeneity [Baron et al., 2016, Hendley et al., 2021].

Within the original endothelial annotation, RAG identified a marker-supported post-capillary venule-like endothelial cluster 31 marked by Selp/Ackr1/Vwf while retaining endothelial markers Pecam1, Cdh5, Kdr, Flt1, Tek, and Plvap. The additional enrichment of Madcam1, Lrg1, and Sele supports a venous/adhesion-associated programme, consistent with single-cell endothelial atlases describing venous and post-capillary venule endothelial heterogeneity [Kalucka et al., 2020, Wakabayashi and Naito, 2023]. These results indicate that RAG can recover annotated rare or non-endocrine pancreatic populations and reveal ductal and endothelial substructure within major pancreatic annotations.

In addition to the ductal and endothelial candidate subpopulations reported in the main text, RAG identified additional candidate marker-supported states within the beta and macrophage annotations of the mouse pancreas dataset. The corresponding UMAP overview and marker-supported extra-cluster violin plot are provided in Supplementary Figs. S10 and S12.

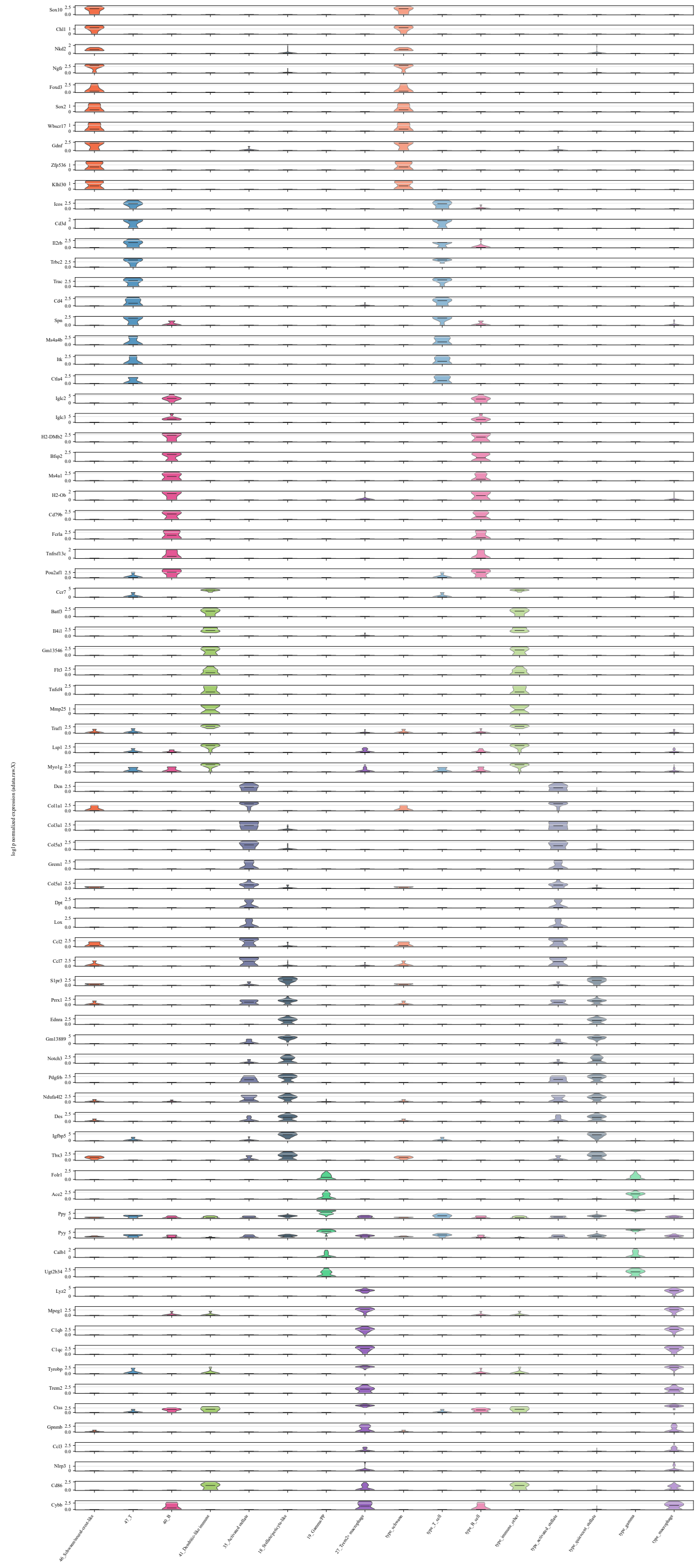

Figure S11: Marker-gene validation of RAG-recovered known annotated small clusters in the mouse pancreas dataset.

Alt text: Violin plots showing representative marker-gene expression for known annotated small clusters and their corresponding original annotation references in the mouse pancreas dataset.

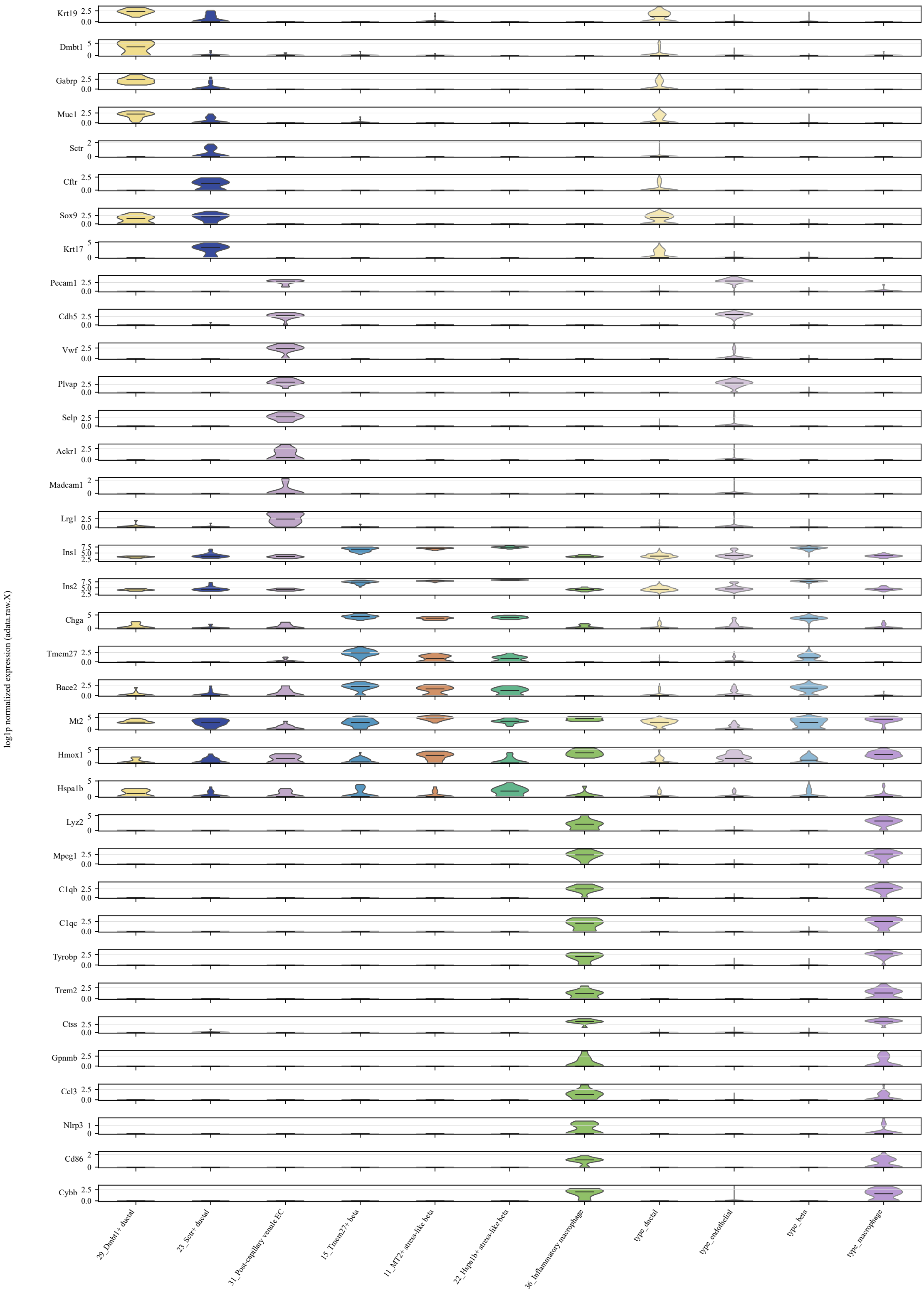

Figure S12: Marker-gene validation of RAG-derived marker-supported extra subpopulations in the mouse pancreas dataset.

Alt text: Violin plots showing representative marker-gene expression for marker-supported extra clusters and their corresponding original annotation references in the mouse pancreas dataset.

Within the original beta-cell annotation, RAG separated three candidate beta-associated states with marker support. Cluster 15 showed a Chga/Tmem27/Bace2-positive endocrine signature, suggesting a possible functional beta-associated state. Cluster 11 was enriched for Mt2, Bnip3, and Hmox1, suggesting an oxidative-stress or metal-response-like beta-associated programme. Cluster 22 was enriched for Dnaja4, Dnajc24, Derl3, and Hspa1b, suggesting a heat-shock/ER-proteostasis-like beta-associated programme. These candidate interpretations are consistent with prior evidence linking Tmem27/Bace2 to beta-cell function, oxidative-stress and hypoxia responses to beta-cell dysfunction, and ER proteostasis to beta-cell stress and failure [Esterhazy et al., 2011, Gerber and Rutter, 2017, Yong et al., 2021]. Because stress-response and heat-shock signatures can also arise from isolation or dissociation-related perturbation, clusters 11 and 22 were interpreted conservatively as stress-like beta-associated states.

Within the macrophage annotation, RAG separated a canonical macrophage cluster and a candidate activated/inflammatory macrophage-like state. Cluster 27 largely recovered the canonical macrophage programme, marked by Lyz2/C1qb/C1qc/Tyrobp/Mpeg1, together with Trem2 and Ctss. Cluster 36 retained macrophage/myeloid identity but showed additional inflammatory activation markers, including Ccl3/Nlrp3/Cd86/Clec4e/Cst7/Cybb. Thus, cluster 27 was interpreted as recovery of the reference macrophage programme, whereas cluster 36 was conservatively interpreted as a candidate activated or inflammatory macrophage-like state [Baron et al., 2016, Willemsen and de Winther, 2020].

Several additional splits within abundant endocrine or epithelial annotations were not retained as high-confidence biological subpopulations. In particular, multiple alpha, delta, beta, ductal, and gamma/PP splits largely preserved the same reference marker programme without a coherent additional biological signature, and were therefore treated as original-annotation over-segmentation rather than independent marker-supported states. This conservative filtering ensured that only clusters with both retained cell-type identity and an interpretable additional marker programme were reported as candidate biologically meaningful subpopulations.

Human pancreas

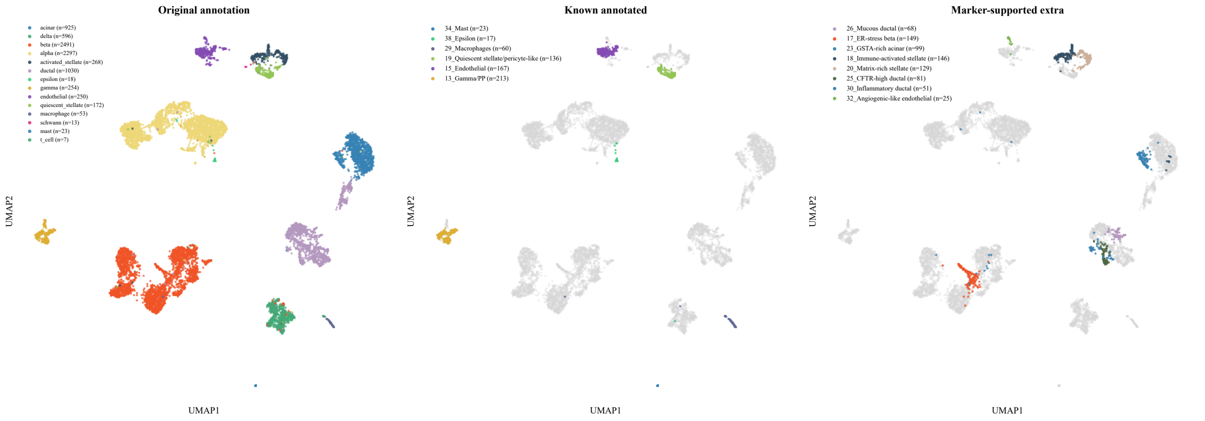

Figure S13: UMAP visualisation of RAG-derived marker-supported small clusters in the human pancreas dataset.

Alt text: UMAP panels showing original annotations, known annotated small clusters, and marker-supported extra clusters in the human pancreas dataset.

Human pancreas dataset: known annotated small clusters. In the human pancreas dataset, RAG recovered known rare or non-endocrine pancreatic populations as independent clusters, including mast cells, epsilon cells, macrophages, quiescent stellate cells, endothelial cells, and gamma/PP cells, consistent with published human pancreas single-cell atlases and curated marker resources [Baron et al., 2016, Muraro et al., 2016, Hu et al., 2023]. These recovered clusters showed canonical marker signatures, including TPSAB1/KIT/CPA3 for mast cells, GHRL for epsilon cells, CD163/CD68 for macrophages, PDGFRB/ADIRF/FABP4 for quiescent stellate cells, VWF/PECAM1/CD34 for endothelial cells, and PPY for gamma/PP cells. The corresponding UMAP overview and known-cluster violin plot are provided in Supplementary Figs. S13 and S14.

Human pancreas dataset: marker-supported small clusters. RAG also separated marker-supported subpopulations from major pancreatic annotations.

Within the ductal compartment, RAG resolved a putative mucous-secretion-associated ductal cluster 26 marked by TFF1/TFF2 together with AGR2/AGR3, while retaining ductal markers KRT19/KRT7. This pattern is consistent with the TFF1/TFF2-enriched mucous-secretion-related ductal programme described in GSE84133[Baron et al., 2016].

Within the beta-cell annotation, RAG identified a putative ER-stress-associated beta-cell cluster 17 marked by DDIT3/HERPUD1 together with UPR-related genes TRIB3/DNAJB9, while retaining the beta-cell identity marker IAPP, consistent with the beta-cell ER-stress heterogeneity reported in GSE84133[Baron et al., 2016].

Within the acinar compartment, RAG identified a putative GSTA-rich acinar cluster 23 marked by canonical digestive-enzyme genes CEL/PNLIP/CPA2 together with GSTA1/GSTA2/MGST1. This pattern supports an acinar identity and suggests a glutathione/redox-associated acinar phenotype, consistent with pancreatic acinar marker programmes and prior evidence of acinar transcriptional heterogeneity and redox-linked stress responses [Baron et al., 2016, Muraro et al., 2016, Pan et al., 2023].

These results indicate that RAG can recover annotated rare or non-endocrine pancreatic populations and reveal acinar, ductal, and beta-cell substructure within major pancreatic annotations.

In addition to the candidate marker-supported acinar, ductal, and beta-cell subpopulations reported in the main text, RAG identified additional candidate substructures within the activated-stellate, ductal, and endothelial annotations of the GSE84133 human pancreas dataset. The corresponding UMAP plots and violin plots of gene expression patterns are provided in Supplementary Figs. S13 and S15.

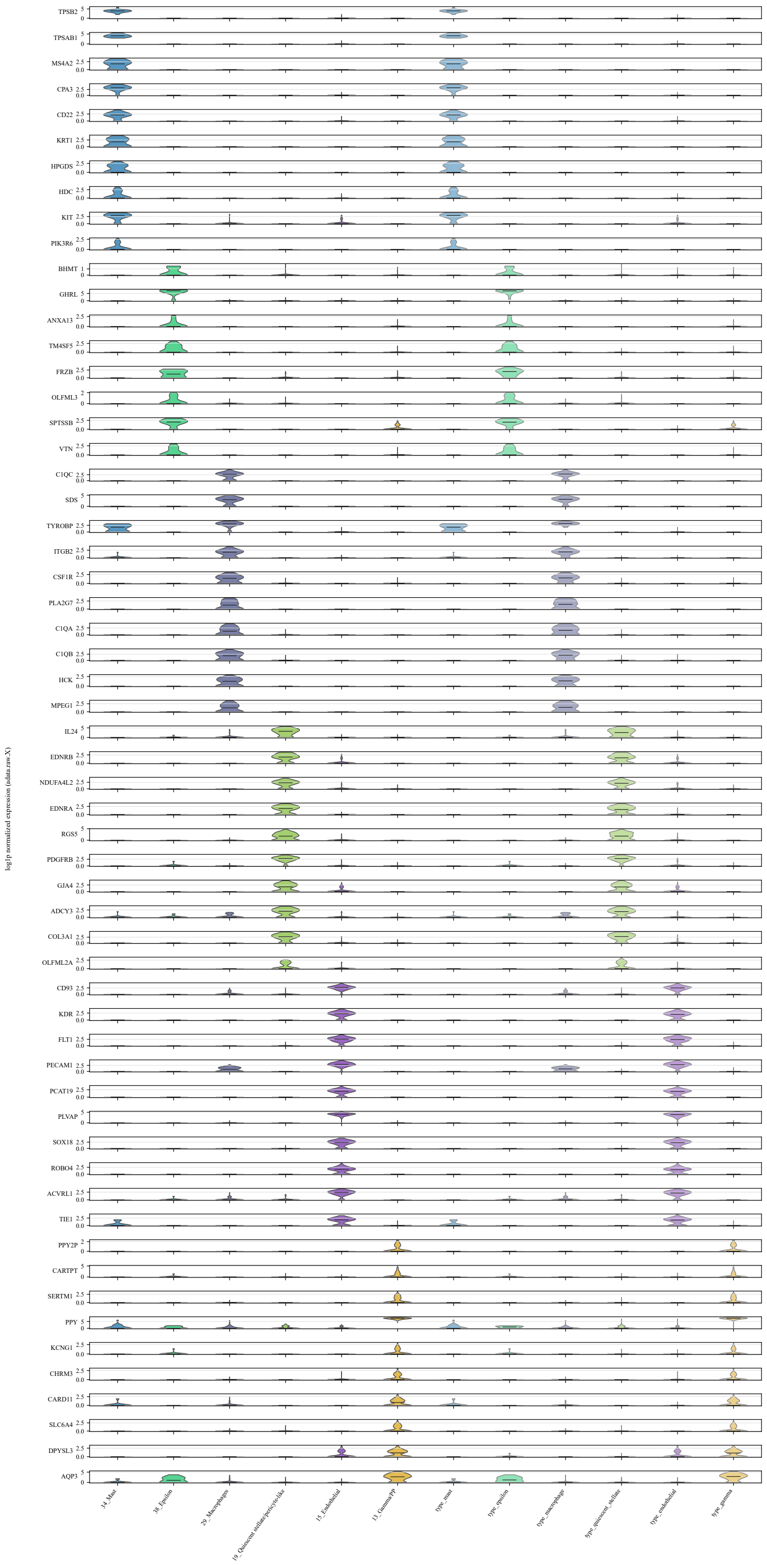

Figure S14: Marker-gene validation of RAG-recovered known annotated small clusters in the human pancreas dataset.

Alt text: Violin plots showing representative marker-gene expression for known annotated small clusters and their corresponding original annotation references in the human pancreas dataset.

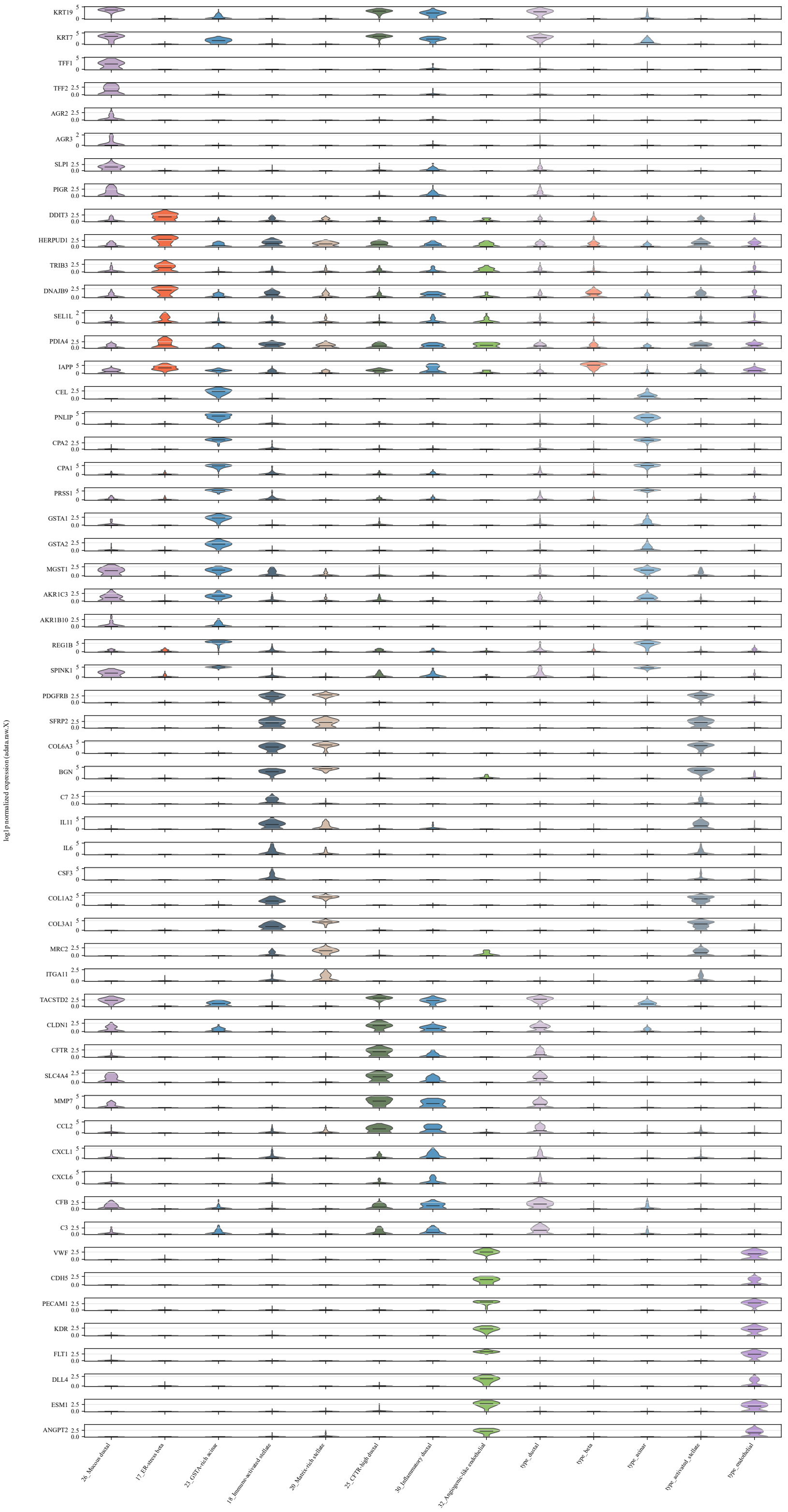

Figure S15: Marker-gene validation of RAG-derived marker-supported extra subpopulations in the human pancreas dataset.

Alt text: Violin plots showing representative marker-gene expression for marker-supported extra clusters and their corresponding original annotation references in the human pancreas dataset.

Within the original activated-stellate annotation, RAG separated two candidate activated-stellate/stromal clusters. Both clusters retained shared stellate and stromal markers, including PDGFRB, SFRP2, COL6A2, COL6A3, BGN, VCAN, and MMP2. Cluster 18 showed a cytokine/complement-associated activated-stromal signature, with elevated C7, IL11, IL6, CSF3, APOD, and PTGDS, whereas cluster 20 showed stronger enrichment of matrix-remodelling and collagen-associated genes, including COL1A2, COL3A1, FN1, COL12A1, COL5A1, MRC2, and ITGA11. These patterns are compatible with the extracellular-matrix-related and immune-activated modes of stellate-cell activation described in the original GSE84133 atlas [Baron et al., 2016].

Within the original ductal annotation, RAG further identified candidate marker-supported ductal epithelial states beyond the main-text TFF/AGR-rich cluster. Cluster 25 showed a CFTR-high activated/transport-associated ductal signature, retaining KRT7, KRT19, TACSTD2, CLDN1 together with CFTR, MMP7, SLC4A4, CTGF, and CYR61. Cluster 30 retained ductal epithelial markers KRT7, KRT19, TACSTD2, CLDN1 and showed a more inflammatory ductal programme, including CCL2, CXCL1, CXCL6, CXCL8, CFB, C3, SERPING1, and VCAM1. These clusters were therefore interpreted as candidate ductal epithelial states with marker support, consistent with reported pancreatic ductal heterogeneity [Baron et al., 2016, Hendley et al., 2021].

Within the original endothelial annotation, RAG identified a small candidate angiogenic-like endothelial cluster with marker support. Cluster 32 retained a clear endothelial identity, marked by VWF, CDH5, PECAM1, ESAM, KDR, FLT1, and ROBO4, and showed additional enrichment of angiogenesis- and sprouting-associated genes, including DLL4, ESM1, ANGPT2, NOTCH4, PGF, CXCR4, and FSCN1. This pattern is compatible with endothelial-cell annotation in the original GSE84133 atlas and with prior evidence linking DLL4–Notch signalling and angiogenic EC programmes to sprouting endothelial states [Baron et al., 2016, Hellström et al., 2007, Kalucka et al., 2020].

Several additional splits within abundant endocrine, ductal, or endothelial annotations were not retained as biological subpopulations. In particular, multiple beta, alpha, delta, ductal, endothelial, and gamma-derived splits either preserved the same reference marker programme without a coherent additional biological signature.

S6 Differential Expression Analysis Illustration

Differential expression (DE) analysis was performed in a one-versus-rest manner for each cluster using Scanpy (`sc.tl.rank_genes_groups`; Wilcoxon rank-sum test; `pts=True`, `use_raw=False`, `tie_correct=True`). Clusters with $\leq 0.1\%$ of total cells were excluded before DE testing. Upregulated markers were defined as genes with adjusted p-value (FDR) ≤ 0.05, $\log_2$ fold change ≥ 2, and detection-rate difference ($\text{pct\_in} - \text{pct\_bg}$) ≥ 0.20; clusters with at least 5 such genes were considered meaningful.

References

Maayan Baron, Adrian Veres, Samuel L Wolock, Aubrey L Faust, Renaud Gaujoux, Amedeo Vetere, Jennifer Hyoje Ryu, Bridget K Wagner, Shai S Shen-Orr, Allon M Klein, et al. A single-cell transcriptomic map of the human and mouse pancreas reveals inter-and intra-cell population structure. *Cell systems*, 3(4):346–360, 2016.

Congxue Hu, Tengyue Li, Yingqi Xu, Xinxin Zhang, Feng Li, Jing Bai, Jing Chen, Wenqi Jiang, Kaiyue Yang, Qi Ou, et al. Cellmarker 2.0: an updated database of manually curated cell markers in human/mouse and web tools based on scrna-seq data. *Nucleic acids research*, 51(D1):D870–D876, 2023.

Audrey M Hendley, Arjun A Rao, Laura Leonhardt, Sudipta Ashe, Jennifer A Smith, Simone Giacometti, Xianlu L Peng, Honglin Jiang, David I Berrios, Mathias Pawlak, et al. Single-cell transcriptome analysis defines heterogeneity of the murine pancreatic ductal tree. *Elife*, 10:e67776, 2021.

Joanna Kalucka, Laura PMH de Rooij, Jermaine Goveia, Katerina Rohlenova, Sébastien J Dumas, Elda Meta, Nadine V Conchinha, Federico Taverna, Laure-Anne Teuwen, Koen Veys, et al. Single-cell transcriptome atlas of murine endothelial cells. *Cell*, 180(4):764–779, 2020.

Taku Wakabayashi and Hisamichi Naito. Cellular heterogeneity and stem cells of vascular endothelial cells in blood vessel formation and homeostasis: Insights from single-cell rna sequencing. *Frontiers in Cell and Developmental Biology*, 11:1146399, 2023.

Daria Esterhazy, Ina Stützer, Haiyan Wang, Markus P Rechsteiner, Jeremy Beauchamp, Heinz Döbeli, Hans Hilpert, Hugues Matile, Michael Prummer, Alexander Schmidt, et al. Bace2 is a β cell-enriched protease that regulates pancreatic β cell function and mass. *Cell metabolism*, 14(3):365–377, 2011.

Philipp A Gerber and Guy A Rutter. The role of oxidative stress and hypoxia in pancreatic beta-cell dysfunction in diabetes mellitus. *Antioxidants & redox signaling*, 26(10):501–518, 2017.

Jing Yong, James D Johnson, Peter Arvan, Jaeseok Han, and Randal J Kaufman. Therapeutic opportunities for pancreatic β-cell er stress in diabetes mellitus. *Nature Reviews Endocrinology*, 17(8): 455–467, 2021.

Lisa Willemsen and Menno PJ de Winther. Macrophage subsets in atherosclerosis as defined by single-cell technologies. *The Journal of pathology*, 250(5):705–714, 2020.

Mauro J Muraro, Gitanjali Dharmadhikari, Dominic Grün, Nathalie Groen, Tim Dielen, Erik Jansen, Leon Van Gurp, Marten A Engelse, Francoise Carlotti, Eelco Jp De Koning, et al. A single-cell transcriptome atlas of the human pancreas. *Cell systems*, 3(4):385–394, 2016.

Zhaolong Pan, Jan-Lars Van den Bossche, Eva Rodriguez-Aznar, Pauline Janssen, Olaya Lara, Gamze Ates, Ann Massie, Diedert Luc De Paep, Isabelle Houbracken, Marco Mambretti, et al. Pancreatic acinar cell fate relies on system xc-to prevent ferroptosis during stress. *Cell Death & Disease*, 14(8): 536, 2023.

Mats Hellström, Li-Kun Phng, Jennifer J Hofmann, Elisabet Wallgard, Leigh Coultas, Per Lindblom, Jackelyn Alva, Ann-Katrin Nilsson, Linda Karlsson, Nicholas Gaiano, et al. Dll4 signalling through notch1 regulates formation of tip cells during angiogenesis. *Nature*, 445(7129):776–780, 2007.
